# Phosphonium Ionic Liquids for Electrochemical Energy Storage: Structure–Property–Interphase Relationships and Design Rule

**DOI:** 10.1002/advs.78370

**Published:** 2026-10-22

**Authors:** Amir Haghipour, Ishanka Thennakoon, Aishwaryaba Jadeja, Daniel Rauber, Markus Gallei, Rolf Hempelmann, Sangwon Kim, Jungtae Kim

**Affiliations:** ^1^ Korea Institute of Science and Technology (KIST) Europe Saarbrücken Germany; ^2^ Saarland University Polymer Chemistry Saarbrücken Germany; ^3^ Saarene, Saarland Center for Energy Materials and Sustainability Saarbrücken Germany; ^4^ Transfercenter Sustainable Electrochemistry Saarland University Saarbrücken Germany

**Keywords:** deep eutectic solvents, electrolyte design, interphase engineering, metal batteries, phosphonium ionic liquids, post‐lithium energy storage, supercapacitors, sustainable electrolytes

## Abstract

Phosphonium ionic liquids are emerging electrolyte platforms for safer and more durable electrochemical energy‐storage systems because they combine low volatility, reduced flammability, thermal robustness, broad electrochemical stability windows, and highly tunable cation–anion chemistry. Their performance emerges from coupled cation–anion structure, salt concentration, electrolyte formulation, solvation environment, and electrode chemistry. This Review develops a structure–property–interphase framework for phosphonium‐based electrolytes in lithium, sodium, zinc, magnesium, and aluminum batteries, as well as supercapacitors and quasi‐solid‐state systems. For phosphonium‐based electrolytes, we analyze how alkyl chain length, branching, ether functionalization, and anion identity govern viscosity, conductivity, ion coordination, metal plating/stripping, corrosion, gas evolution, and solid–electrolyte and cathode–electrolyte interphase formation. The analysis shows that smaller, asymmetric, branched, or ether‐functionalized cations generally promote faster ion transport, whereas long‐chain hydrophobic cations are better suited for thermally robust, low‐volatility, gel, or deep‐eutectic‐type formulations. Finally, we propose practical design rules emphasizing low‐viscosity cation design, fluorine‐reduced anion chemistry, standardized benchmarking, full‐cell validation, and surface‐sensitive/operando interphase characterization.

## Graphical abstract

Phosphonium ionic liquids offer a versatile platform for safer and more durable energy storage. This Review connects cation–anion structure with solvation, ion transport, electrochemical stability, and interphase formation across diverse battery chemistries. These structure–property relationships provide design principles for engineering functional interfaces and developing application‐specific electrolytes for advanced energy‐storage systems.

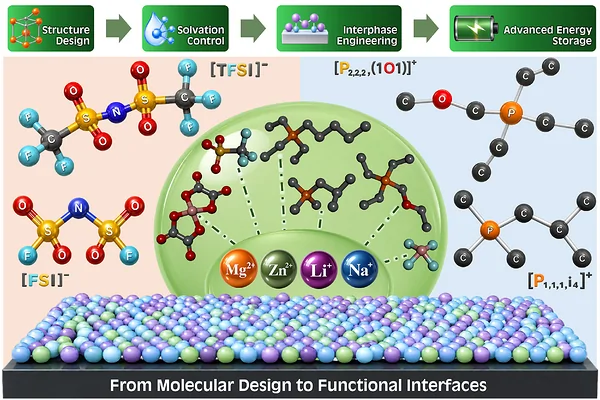

**Abbreviations**
Al: Aluminum
AN: Acetonitrile
ATR‐FTIR: Attenuated total reflection Fourier‐transform infrared spectroscopy
BF_4_
: Tetrafluoroborate
BOB: Bis(oxalato)borate
CE: Coulombic efficiency
CEI: Cathode–electrolyte interphase
cryo‐ET: Cryogenic electron tomography
cryo‐TEM: Cryogenic transmission electron microscopy
DCA: Dicyanamide
DEC: Diethyl carbonate
DES: Deep eutectic solvent
DME: 1,2‐Dimethoxyethane
DTG: Derivative thermogravimetry
EC: Ethylene carbonate
EC‐AFM: Electrochemical atomic force microscopy
EES: Electrochemical energy storage
EIS: Electrochemical impedance spectroscopy
EMC: Ethyl methyl carbonate
EQCM: Electrochemical quartz crystal microbalance
EQCM‐D: Electrochemical quartz crystal microbalance with dissipation monitoring
ESW: Electrochemical stability window
ESS: Energy‐storage system
FAP: Tris(pentafluoroethyl)trifluorophosphate
FSI: Bis(fluorosulfonyl)imide
FTIR: Fourier‐transform infrared spectroscopy
GBL: γ‐Butyrolactone
HER: Hydrogen evolution reaction
IL: Ionic liquid
IR: Infrared
LHCE: Localized high‐concentration electrolyte
LIB: Lithium‐ion battery
LiFSI: Lithium bis(fluorosulfonyl)imide
LFP: Lithium iron phosphate
NaFSI: Sodium bis(fluorosulfonyl)imide
PC: Propylene carbonate
PEDOT: poly(3,4‐ethylenedioxythiophene)
PF_6_
: Hexafluorophosphate
pkHRR: Peak heat release rate
PN: Propionitrile
PM‐IRRAS: Polarization‐modulation infrared reflection–absorption spectroscopy
SEI: Solid–electrolyte interphase
SERS: Surface‐enhanced Raman spectroscopy
SEIRAS: Surface‐enhanced infrared absorption spectroscopy
SIB: Sodium‐ion battery
TCM: Tricyanomethanide
*T*_dec_
: Reported thermal decomposition temperature, with the criterion specified in the original source
TFA: Trifluoroacetate
TGA: Thermogravimetric analysis
THF: Tetrahydrofuran
THR: Total heat release
*T*_10%_
: Temperature at 10% mass loss
*T*_onset_
: Thermal decomposition onset temperature
*T*_max_
: Temperature of maximum decomposition rate
ToF‐SIMS: Time‐of‐flight secondary ion mass spectrometry
TTI: Time to ignition
WiS: Water‐in‐salt
XPS: X‐ray photoelectron spectroscopy
ZIB: Zinc‐ion battery
*: Chemical formulas, phosphonium cation shorthand (e.g., [P_2,2,2,5_]^+^, [P_2,2,2_,(1O1)]^+^), and anion formulas (e.g., [FSI]^−^, [TFSI]^−^) are defined in Table 1 and the relevant figure captions and are therefore not repeated here.

## 1 Introduction

### 1.1 Electrolyte Limitations in Next‐Generation ESS

EES is central to electrified transportation, renewable‐energy integration, and grid‐scale storage, with global battery demand expected to increase substantially by 2030 [1, 2, 3, 4, 5]. LIBs currently dominate this landscape because of their high energy density, mature manufacturing base, high round‐trip efficiency, and broad deployment in portable electronics, electric vehicles, and stationary storage [2, 3, 4, 5]. However, their long‐term sustainability and safety remain constrained by critical‐metal supply risks, flammable carbonate‐based electrolytes, thermal instability under abuse conditions, transition‐metal dissolution from cathode active materials, and the need for lower‐cost post‐lithium chemistries [2, 3, 4, 5]. These concerns have intensified interest in sodium‐, zinc‐, magnesium‐, and aluminum‐based batteries, which rely on more abundant elements and are attractive for large‐scale energy storage [6, 7, 8, 9, 10].

Despite this promise, the practical development of post‐lithium systems remains strongly limited by electrolyte chemistry [11, 12, 13]. An ideal electrolyte must simultaneously provide high ionic conductivity, a wide ESW, low volatility and flammability, high thermal stability, and stable interfacial compatibility with reactive metal anodes and high‐voltage cathodes [4, 5, 8, 14]. Conventional carbonate‐, ether‐, or nitrile‐based electrolytes often fail to meet these requirements because of limited oxidative/reductive stability, decomposition‐induced gas evolution, parasitic interfacial reactions, and safety concerns associated with volatile and flammable components [4, 5, 15, 16]. In multivalent systems in particular, strong cation–solvent coordination further increases desolvation barriers and slows charge‐transfer kinetics, while poorly conductive or passivating interphases can suppress reversible metal plating/stripping [8, 9, 17].

ILs have therefore emerged as promising electrolyte platforms for safer energy‐storage devices [18, 19, 20]. Their negligible vapor pressure, low flammability, thermal robustness, and modular cation–anion chemistry enable molecular‐level control over viscosity, ionic conductivity, ion coordination, electrochemical stability, and interfacial reactivity [3, 4, 14, 21]. Beyond neat liquid salts, related formats such as ionogels, solvate ILs, concentrated salt–solvent mixtures, hybrid inorganic–organic electrolytes, polymeric ILs, and deep‐eutectic‐type systems have further expanded the electrolyte‐design space [3, 4, 22, 23, 24, 25]. Nitrogen‐centered ILs, including imidazolium, ammonium, pyrrolidinium, and piperidinium systems, still face trade‐offs among transport, electrochemical stability, thermal robustness, and interfacial compatibility [26, 27, 28, 29]. Imidazolium ILs often show favorable conductivity but limited reductive stability, whereas pyrrolidinium, piperidinium, and ammonium ILs generally offer broader apparent ESWs at the cost of higher viscosity or stronger ion association [26, 27, 28].

These limitations have motivated increasing interest in phosphonium ILs, particularly compact, asymmetric, or ether‐functionalized cations that can combine broad apparent ESWs, improved transport, lower melting tendency, and high thermal robustness [21, 26, 28, 30, 31]. Compared with imidazolium systems, phosphonium ILs generally provide improved reductive stability; compared with many pyridinium ILs, they often exhibit lower melting points and higher thermal stability; and compared with many ammonium analogues, appropriately designed phosphonium cations can reduce viscosity and enhance ion transport [21, 26, 27, 28, 30, 31, 32]. However, these advantages are system‐dependent and governed by cation architecture, anion identity, salt concentration, impurity level, and electrolyte formulation [21, 26, 27, 28, 29, 30, 31]. These features establish phosphonium ILs as a distinct and highly tunable platform for electrolyte design.

### 1.2 Why Phosphonium ILs? Molecular Origin of Their Distinct Electrolyte Behavior

At the molecular level, phosphonium ILs provide a distinct electrochemical environment because the bulky tetraalkylphosphonium cation can weaken localized cation–anion interactions, reduce direct coordination to reactive anions, and modify ion‐domain organization [27, 30, 33]. This behavior is particularly relevant for short‐chain, asymmetric, or branched phosphonium cations, where lower viscosity, improved ion dissociation, and altered interfacial adsorption can influence both bulk transport and electrode‐surface chemistry [4, 28, 30, 34, 35, 36]. Phosphonium bis(fluorosulfonyl)imide ([FSI]¯) systems have been associated with enhanced ionic conductivity in hybrid carbonate electrolytes, reversible oxygen redox chemistry in Na–O_2_ electrolytes, and the formation of phosphorus‐containing SEI components on graphite–silicon (Gr/Si) composite anodes [37]. Molecular simulation and transport studies further show that side‐chain length, cation symmetry, water content, and anion coordination affect local ion organization, diffusion coefficients, viscosity, and ionic conductivity in phosphonium ILs [15, 26, 28, 34, 35].

This distinction is illustrated in Figure 1, which compares reported distributions of apparent ESW across IL cation families [38, 39, 40, 41, 42, 43, 44, 45, 46, 47, 48, 49, 50, 51, 52, 53, 54, 55, 56, 57, 58, 59, 60, 61, 62]. Apparent ESWs reported in the literature depend strongly on experimental conditions, including electrode material, reference scale, scan rate, temperature, impurity level, and cutoff‐current criterion [4, 30, 63, 64]. Accordingly, the ESW values discussed throughout this review should be interpreted as comparative literature descriptors rather than intrinsic material constants [31, 65]. The broad spread within each cation family emphasizes that apparent ESW is controlled as much by electrolyte formulation as by cation identity [31, 65]. Phosphonium‐based ILs and phosphonium‐containing polymeric ILs have frequently been reported to exhibit higher thermal stability and greater chemical robustness than related nitrogen‐centered analogues, although the extent of this advantage depends strongly on cation structure, anion chemistry, and operating conditions [31, 65, 66, 67].

**FIGURE 1:**
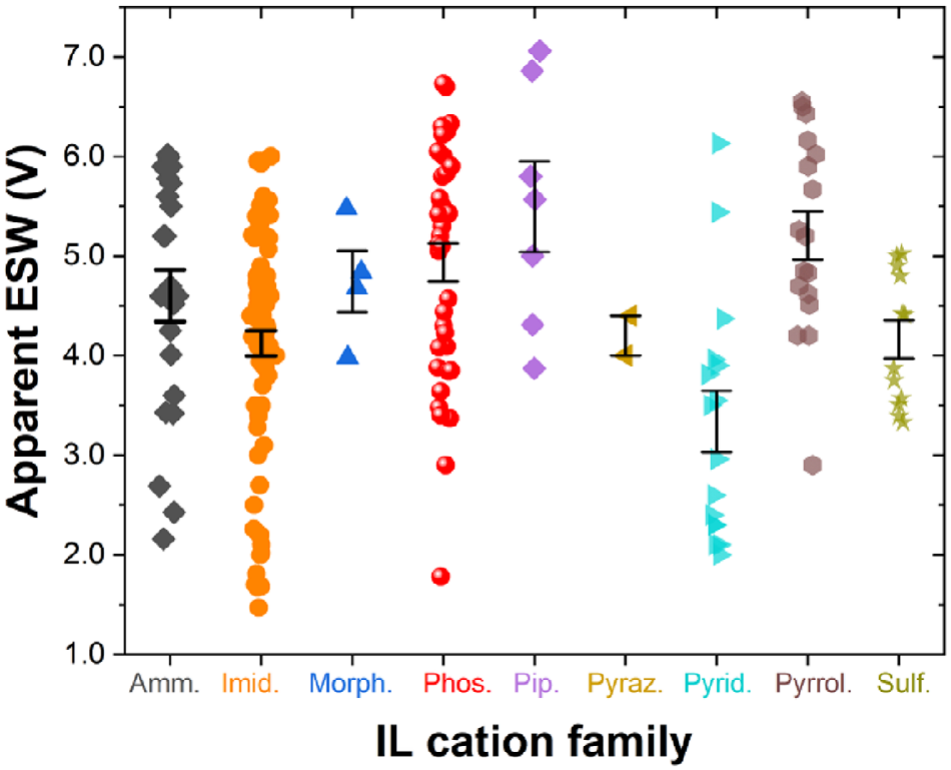
Literature‐level distribution of apparent electrochemical stability windows (ESWs) for ILs grouped by cation family. Individual points represent reported apparent ESW values for ILs classified as Amm. = ammonium; Imid. = imidazolium; Morph. = morpholinium; Phos. = phosphonium; Pip. = piperidinium; Pyraz. = pyrazolium; Pyrid. = pyridinium; Pyrrol. = pyrrolidinium; and Sulf. = sulfonium‐based systems. Horizontal bars and error bars indicate the arithmetic mean ± standard error of the mean (SEM) within each cation‐family group. The number of data points (*n*) differs among cation families according to the available literature data. Because the compiled values include different anions, salt concentrations, electrolyte compositions, electrode configurations, scan rates, temperatures, impurity levels, and cut‐off‐current criteria, the figure should be interpreted as a descriptive literature‐level distribution rather than a direct ranking of intrinsic cation stability. In particular, lower or broader ESW distributions for some cation families may reflect associated anion chemistry or electrolyte formulation rather than intrinsic cation instability. Full data and source references are provided in Table S1. The figure was constructed from literature‐reported apparent ESW values compiled in this work [38, 39, 40, 41, 42, 43, 44, 45, 46, 47, 48, 49, 50, 51, 52, 53, 54, 55, 56, 57, 58, 59, 60, 61, 62].

Thermal stability is therefore discussed separately below, along with flammability and the effects of measurement protocol [14, 68, 69]. These molecular features make phosphonium ILs attractive for energy‐storage systems, especially where high ionic conductivity, broad apparent ESWs, low volatility, improved safety, and controlled metal–electrolyte interfaces are required [4, 15, 28]. Reported examples include improved high‐voltage cathode compatibility in Li‐ion cells, Li‐metal and anode‐free cycling [FSI]¯ electrolytes, and stable Na‐metal cycling in concentrated phosphonium [FSI]^−^ systems [10, 15, 70, 71]. These coupled benefits and limitations motivate a structure–property–interphase analysis that connects molecular design, transport behavior, apparent ESW, and electrode‐specific interfacial chemistry [4, 10, 15, 30, 63, 64, 70, 71].

### 1.3 Knowledge Gaps and Motivation for This Review

Despite growing interest in phosphonium ILs, their role in EES remains less systematically established than that of imidazolium‐, ammonium‐, pyrrolidinium‐, and piperidinium‐based ILs [72, 73, 74]. Reported studies have demonstrated promising behavior in lithium, sodium, zinc, magnesium, and aluminum batteries, as well as in supercapacitors, hybrid gels, deep‐eutectic‐type electrolytes, and polymeric IL systems [17, 75, 76, 77, 78]. These advances remain difficult to generalize because bulk transport, interfacial reactivity, and testing protocol are strongly coupled [30, 79, 80]. The challenge is therefore not a shortage of individual phosphonium‐IL studies, but the difficulty of translating fragmented results into generalizable relationships among molecular structure, ion transport, apparent electrochemical stability, metal compatibility, corrosion, and solid–electrolyte and cathode–electrolyte interphase (SEI/CEI) formation [81, 82].

Individual reports indicate that short‐chain, asymmetric, branched, and ether‐functionalized phosphonium cations can reduce viscosity and improve ion mobility, yet these effects are rarely systematically benchmarked across different anion families, electrolyte formats, and battery chemistries [26, 27, 30, 34]. Anions such as [FSI]¯, dicyanamide [DCA]¯, tetrafluoroborate [BF_4_]¯, bis(trifluoromethanesulfonyl)imide [TFSI]¯, trifluoromethanesulfonate [OTf]¯, and bis(oxalato)borate [BOB]¯ govern not only viscosity and ionic conductivity, but also salt dissociation, oxidative/reductive stability, metal plating/stripping, corrosion suppression, gas evolution, and interphase chemistry [83, 84]. Thus, phosphonium ILs should be evaluated not only as electrolyte media, but as molecularly tunable bulk–interfacial platforms whose function depends on the target electrode chemistry [4, 30, 81, 84].

Previous reviews have addressed important but narrower aspects of phosphonium ionic liquids. Earlier work has focused primarily on structure–conductivity relationships in phosphonium ILs and poly(ionic liquid)s [25, 31], while subsequent reviews have emphasized phosphonium‐based polymer electrolytes for lithium‐ion batteries [25, 85]. Other reviews have focused on individual phosphonium cation families or broader applications of phosphonium ILs [4, 21, 86]. More general reviews of ionic‐liquid electrolytes for energy storage typically treat phosphonium systems as one cation class among several [4, 87, 88]. Consequently, a cross‐chemistry analysis that explicitly connects phosphonium‐cation architecture and anion chemistry with solvation, ion transport, protocol‐dependent electrochemical and thermal stability, dynamic interphase behavior, and application‐specific performance remains lacking [4, 21, 25, 31, 81, 85, 87, 88].

This Review addresses this gap through a phosphonium‐specific structure–property–interphase framework spanning lithium, sodium, zinc, magnesium, and aluminum batteries, as well as supercapacitors and quasi‐solid‐state systems. By combining literature benchmarking with mechanistic analysis of cation–anion interactions, transport, SEI/CEI chemistry, corrosion, and electrode compatibility, we derive transferable design rules and identify the experimental validation required to distinguish intrinsic molecular effects from formulation‐ and protocol‐dependent behavior.

## 2 Structure–Property Relationships of Phosphonium ILs

### 2.1 Molecular Design Framework

Phosphonium ILs retain the general modularity of ILs, yet their structure–property relationships are strongly governed by the architecture of the tetraalkylphosphonium cation and its interaction with the counter anion [30, 89]. Variations in alkyl‐chain length, branching, cation symmetry, functionalization, and anion identity alter cation–anion association, local ion organization, charge distribution, and transport dynamics, thereby influencing density, viscosity, ionic conductivity, diffusion coefficients, thermal stability, and interfacial behavior [90, 91]. This tunability enables targeted optimization of transport behavior, electrochemical stability, and interfacial chemistry for different battery chemistries and electrolyte formats [3, 4, 89, 90, 91, 92, 93]. Figure 2 summarizes the relationships among these molecular design variables, electrolyte formats, target chemistries, and validation criteria [3, 4, 89, 93].

**FIGURE 2:**
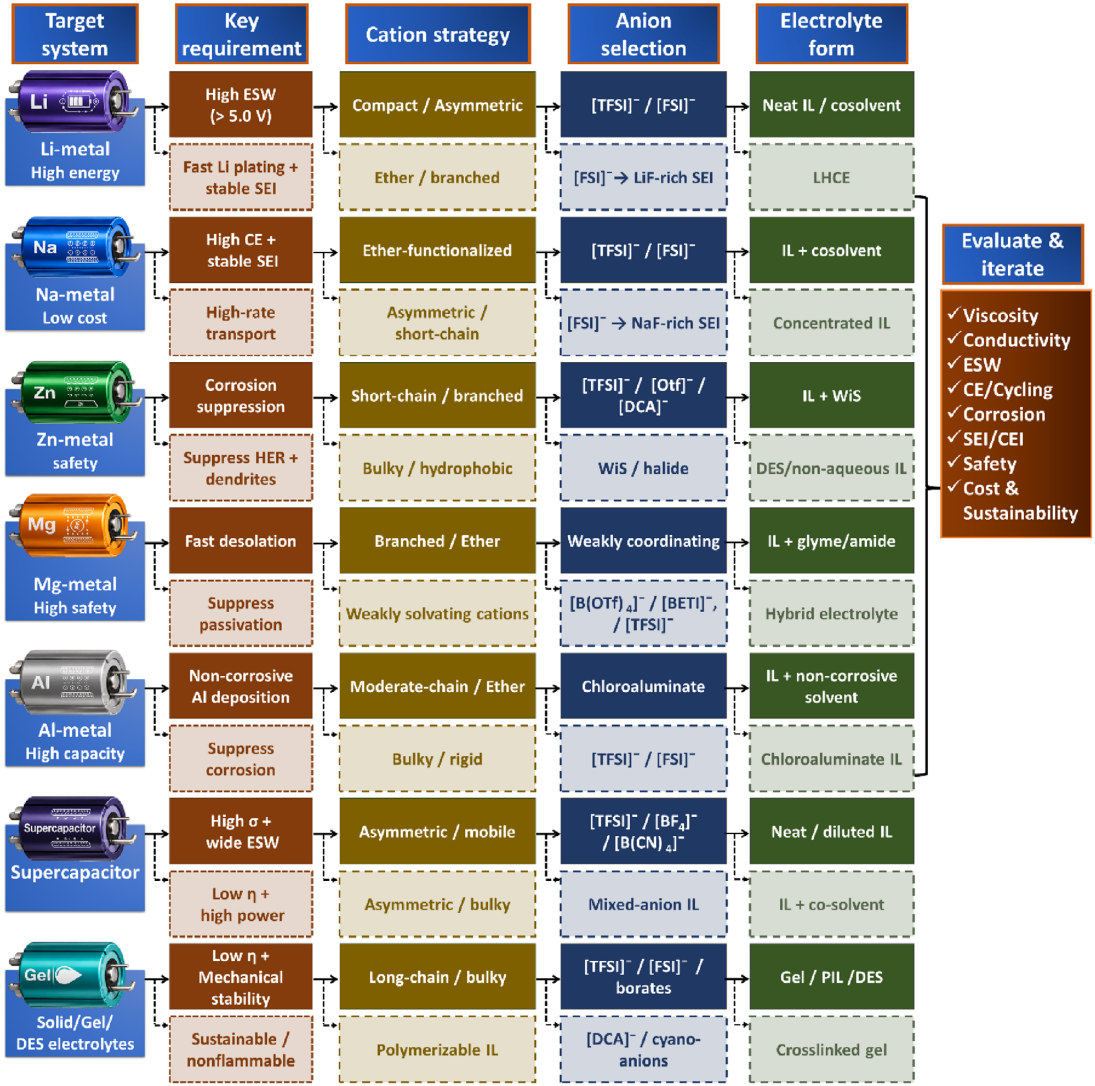
Decision framework for selecting phosphonium IL electrolytes across Li‐, Na‐, Zn‐, Mg‐, and Al‐metal batteries, supercapacitors, and quasi‐solid‐state systems. For each chemistry, the dominant electrochemical requirements are linked to phosphonium‐cation design, anion selection, and electrolyte formulation, followed by practical evaluation of transport, electrochemical stability, interphase behavior, safety, cost, and sustainability. Constructed as a conceptual synthesis from representative literature reports [10, 15, 18, 23, 26, 30, 33, 94, 95, 96, 97, 98, 99, 100]. σ and ƞ denote ionic conductivity and viscosity, respectively. Abbreviations: PIL, poly(ionic liquid); DES, deep eutectic solvent.

Throughout this section, alkyl‐chain composition refers to side‐chain length and branching, whereas electrolyte composition/format denotes neat ILs, salt‐in‐IL electrolytes, hybrid solvent systems, polymer/gel electrolytes, and deep‐eutectic‐type formulations [3, 4, 89, 93]. Together, these parameters define the balance between transport, stability, and interfacial compatibility [26, 30, 101]. Early studies on low‐viscosity phosphonium ILs showed that triethylalkylphosphonium cations paired with [TFSI]¯ can provide lower viscosities and higher conductivities than corresponding ammonium analogues [26, 27, 30]. For example, methoxy‐functionalized phosphonium ILs reached viscosities of 35–44 mPa s at 25°C and broad apparent ESWs under the reported voltammetric conditions [26, 27, 30].

### 2.2 Effect of Cation Architecture

Phosphonium‐cation architecture controls key electrolyte descriptors, including transport properties, apparent ESW, thermal robustness, and interfacial compatibility [28, 102]. Representative physicochemical data and chemical shorthand are summarized in Table 1. Because the reported ESW and *T*_dec_ values were compiled from different studies, they should be interpreted as comparative descriptors rather than absolute operating limits. As discussed in the Introduction, the reported values remain strongly protocol‐dependent [30, 32, 69, 103, 104, 105, 106, 107, 108, 109, 110, 111, 112, 113, 114, 115, 116]. Key cation variables include size, alkyl‐chain composition, symmetry, ether functionalization, and substituent arrangement around the phosphorus center [31, 117, 118, 119]. These factors control ion packing, free volume, cation–anion organization, and segmental mobility, thereby defining the balance between viscosity and ionic conductivity [84, 120, 121, 122].

**TABLE 1:** Representative physicochemical properties of phosphonium ILs as a function of cation architecture and anion identity. Conductivity (*σ*), viscosity (*ƞ*), density (*ρ*), apparent ESW, and *T*_dec_ are shown where available. Conductivity, viscosity, and density values are reported at 25°C unless stated otherwise. Values are protocol‐dependent descriptors. *T*_dec_ = denotes the thermal‐decomposition temperature reported in the original source and is not necessarily an onset temperature.

Cation	Anion	Abbv.	*σ* (mS cm^−^^1^)	*η* (mPa s)	*ρ* (g cm^−^^3^)	ESW (V)	*T*_dec_ (°C)
Triethylpentyl [27, 95, 109, 110]	[FSI]^−^	[P_2,2,2,5_][FSI]	4.63	69	1.22	4.3	371
Triethylpentyl [30, 95, 110]	[TFSI]^−^	[P_2,2,2,5_][TFSI]	2.10	85	1.30	4.9	458
Triethylpentyl [111]	[B(CN)_4_]^−^	[P_2,2,2,5_][B(CN)_4_]	3.10	52	0.95	4.3	420
Trimethyl‐ isobutyl [49]	[FSI]^−^	[P_1,1,1,_i_4_][FSI]	7.30	41	1.30	6	NR
Trimethyl‐ isobutyl [112]	[TFSI]^−^	[P_1,1,1,_i_4_][TFSI]	1.80	110	NR	3.8	NR
Triethyl (methoxymethyl) [30]	[TFSI]^−^	[P_2,2,2_,(1O1)][TFSI]	3	35	1.44	≥5.3	388
Triethyl (2‐methoxyethyl) [30, 95]	[TFSI]^−^	[P_2,2,2_,(2O1)][TFSI]	3.84	48	1.38	4.1	467
Tetra‐*n*‐butyl [113]	[(FH)_2.3_F]^−^	[P_4,4,4,4_][(FH)_2.3_F]	3.70	47	0.95	5.5	257
Tri‐*n*‐butylmethyl [114]	[TFSI]^−^	[P_1,4,4,4_][TFSI]	0.82	200	1.25	5	390
Tri‐n‐butyl‐*n*‐octyl [30]	[TFSI]^−^	[P_4,4,4,8_][TFSI]	0.27	250	1.18	5.2	373
Trihexyl (tetradecyl) [32, 104, 115]	[TFSI]^−^	[P_6,6,6,14_][TFSI]	0.01	378‐413	1.07	4.5	417
Trihexyl (tetradecyl) [69]	[BOB]^−^	[P_6,6,6,14_][BOB]	0.09	513^a^	1	5.5	310^b^
Trihexyl (tetradecyl) [104]	[DCA]^−^	[P_6,6,6,14_][DCA]	0.01	413–461	0.90	3.7	NR
Trihexyl (tetradecyl) [105]	[FAP]^−^	[P_6,6,6,14_][FAP]	0.08	465	1.18	6.3	NR
Trihexyl (tetradecyl) [106]	[HOx]^−^	[P_6,6,6,14_][HOx]	0.02	1110	0.93	5.7	291

Abbreviations: NR, not reported.

^a^ The viscosity of [P6,6,6,14][BOB] was measured at 293 K.

^b^ For [P6,6,6,14][BOB], 310°C corresponds to 5% mass loss (*T*_5%_ = 583 K) at a heating rate of 10 K min^−^^1^ [69].

At the molecular level, these effects arise because phosphonium‐cation structure changes how ions pack and organize in the liquid [26]. Compact, asymmetric, or branched cations tend to disrupt efficient packing and reduce persistent nonpolar‐domain organization, which can increase free volume and facilitate structural relaxation [27]. Ether‐functionalized cations add local polarity and coordination sites that can modify cation–anion interactions and the coordination environment around dissolved metal ions [34]. As a result, cation architecture can influence metal‐ion transport not only through bulk viscosity, but also through changes in ion pairing, aggregation, and local coordination [34, 122]. These effects remain strongly formulation‐dependent because addition of a metal salt can substantially reorganize ion–ion interactions compared with the neat IL [27, 122, 123].

Cations bearing long hydrocarbon substituents, such as trihexyl(tetradecyl)phosphonium ([P_6,6,6,14_]^+^) and tetrabutylphosphonium ([P_4,4,4,4_]^+^), generally increase hydrophobicity, oil and organic‐phase compatibility, low volatility, and thermal robustness [14, 124, 125, 126]. These effects arise from extended nonpolar alkyl domains, reduced lattice‐packing efficiency, lowered melting tendency, and the chemically stable phosphonium framework [126]. However, the same long‐chain substituents can strengthen nano structural segregation, increase viscosity, and slow ion diffusion, thereby reducing ionic conductivity in bulk phosphonium ILs [126, 127, 128, 129, 130, 131]. As a result, long‐alkyl phosphonium ILs are attractive for thermally robust, low‐volatility electrolyte formulations, but they may require dilution, optimized metal‐salt concentration, suitable anion selection, or gel‐ or DES‐based formulations to achieve sufficient ion transport for battery operation [130, 132].

In contrast, appropriately designed shorter, asymmetric, branched, or ether‐functionalized phosphonium cations can improve liquid‐state electrolyte performance by reducing molecular volume, disrupting efficient ion packing, weakening dispersive interactions, and modifying local ion organization [34, 49, 133]. Triethylpentylphosphonium ([P_2,2,2,5_]^+^) and trimethylisobutylphosphonium ([P_1,1,1,_i_4_]^+^), for example, show that compact or branched phosphonium cations, when paired with suitable anions, can form comparatively low‐viscosity ILs while maintaining broad apparent ESWs [30, 49, 133]. Ether functionalization provides an additional route to enhance ion transport [116, 134, 135]. Table 1 further illustrates that conductivity and thermal stability do not necessarily correlate.

Table 1 illustrates differences in ionic conductivity and reported thermal‐decomposition temperatures across phosphonium ILs. However, missing thermal data and differences in measurement criteria limit direct comparisons of thermal stability. Introducing ether‐containing substituents, such as methoxymethyl or methoxyethyl groups, increases conformational flexibility and disrupts cation packing [136, 137, 138]. Ether functionalization introduces local polarity and coordination sites that can modify cation–anion interactions, ion organization, and the coordination environment around dissolved metal ions [136, 139, 140]. However, ether substitution must be optimized carefully because it can alter oxidative stability, metal compatibility, and interfacial decomposition pathways [139, 140]. Therefore, the benefit of ether‐substituted phosphonium cations depends strongly on the paired anion, dissolved metal salt, and operating voltage window [139, 140]. The quantitative data in Table 1 support these trends. Small or asymmetric phosphonium cations such as [P_2,2,2,5_]^+^ and [P_1,1,1,_i_4_]^+^ provide relatively high ionic conductivities of 4.63 and 7.30 mS cm^−1^, respectively, with moderate viscosities of 69 and 41 mPa s (25°C) [27, 95, 116, 134, 135].

In contrast, long‐chain [P_6,6,6,14_]^+^ salts show substantially lower conductivities (≈0.01–0.09 mS cm^−1^) and higher viscosities (378–1110 mPa s) across tris(pentafluoroethyl)trifluorophosphate ([FAP]¯)‐, [TFSI]¯‐, [BOB]¯‐, [DCA]¯‐, and hydrogen oxalate ([HOx]¯)‐based salt systems [32, 69, 103, 104, 105, 106, 141, 142, 143]. This trend is illustrated by [P_2,2,2_,(2O1)][TFSI] reaching 3.84 mS cm^−1^ and 48 mPa s, whereas larger tributyl‐ or trihexyl‐based analogues remain substantially more viscous [30, 95, 114]. Taken together, these data illustrate formulation‐dependent trade‐offs among transport performance, thermal robustness, and interfacial compatibility, rather than a universal inverse relationship between conductivity and thermal stability [35, 107, 143]. Therefore, cation design should be matched to the target application, with lower‐viscosity cations favored for high‐rate metal batteries and long‐chain hydrophobic cations reserved for thermally robust, low‐volatility, gel, or DES‐type formulations [16, 63, 72, 81, 108, 134, 144].

### 2.3 Cation–Anion Control of Ion Association and Electrolyte Properties

The physicochemical and electrochemical behavior of phosphonium ILs is governed by coupled cation–anion interactions rather than by the ions independently [26, 27, 34, 69]. The phosphonium cation mainly influences the substituent environment, including alkyl‐chain composition, symmetry, branching, functional groups, hydrophobicity, and steric shielding, whereas the anion strongly affects charge delocalization, coordination strength, salt solubility, oxidative stability, corrosion behavior, and interphase formation [26, 34, 63, 69]. Overall, phosphonium IL properties emerge from coupled cation–anion interactions that are further modulated by electrolyte composition and operating conditions [27, 69]. Fluorinated anions such as [FSI]¯, [TFSI]¯, [OTf]¯, and [BF_4_]¯ are widely used because they can provide favorable combinations of apparent ESW, ionic conductivity, and compatibility with alkali‐metal salts [4, 27].

[FSI]¯ often yields lower viscosity and higher conductivity than [TFSI]¯ due to its smaller size and weaker cation–anion association, although this transport advantage can be accompanied by reduced thermal or apparent electrochemical stability [28, 76]. [TFSI]¯ generally provides strong oxidative stability, hydrophobicity, and chemical robustness, making it one of the most widely used anions in phosphonium‐based electrolytes for lithium, sodium, and multivalent‐metal systems [15, 26, 27]. Fluorine‐free anions such as [DCA]^−^, [BOB]^−^, carboxylates, orthoborates, and related borate‐based anions are increasingly attractive for reducing fluorinated‐anion content and improving the sustainability profile of phosphonium electrolytes [63, 69, 145]. However, replacing fluorinated anions is not straightforward, as fluorine‐free anions can significantly alter transport properties, apparent ESW, corrosion behavior, interfacial film formation, and impurity sensitivity [69, 145, 146].

For example, borate‐ and orthoborate‐based anions can improve thermal stability and support the use of fluorine‐reduced electrolyte formulations [14, 69, 145]. However, bulky anion structures and impurities introduced during synthesis, ion exchange, hydrolysis, or incomplete purification, including residual halides, alkali‐metal salts, boron‐containing byproducts, and trace transition‐metal species, can increase viscosity, reduce ion mobility, and alter apparent ESW values [14, 69, 145]. Because many high‐performing phosphonium electrolytes rely on fluorinated anions such as [FSI]¯ and [TFSI]¯, transport optimization must increasingly be balanced against sustainability, toxicity, persistence, and regulatory considerations. Therefore, anion selection in phosphonium electrolytes must balance transport efficiency, electrochemical stability, interfacial reactivity, corrosion/passivation behavior, and sustainability [14, 63, 64, 69, 146].

### 2.4 Viscosity–Conductivity Relationship and Walden Analysis

The degree of coupling between viscosity and ionic conductivity is a key transport descriptor for phosphonium ILs [26, 27, 34, 147]. Walden analysis compares molar conductivity with fluidity and is commonly used to assess ion association, correlated ion motion, and effective charge mobility [26, 34]. However, when applied to neat ILs, this interpretation must be treated carefully because ion transport is coupled to free volume, structural relaxation, cation–anion interactions, impurities, dryness, and measurement conditions [26, 27, 34, 148]. Recent dielectric/spectroscopic and modeling studies show that conductivity depends not only on viscosity, but also on alkyl‐chain structure, anion identity, ion– interaction strength, and segmental dynamics [26, 27, 34, 147, 148, 149]. Nevertheless, comparative Walden plots remain useful for identifying whether changes in cation architecture, anion chemistry, or temperature improve charge transport beyond what would be expected from fluidity alone [26, 27, 34, 147, 148, 149].

Figure 3 summarizes literature‐derived viscosity–conductivity data for selected phosphonium ILs. Electrolytes located closer to the ideal KCl line show more efficient charge transport relative to their fluidity, whereas larger downward deviations indicate stronger ion association, correlated ion motion, aggregation, or reduced effective charge mobility [26, 27, 34, 147, 148]. Within the compiled data set, compact or ether‐functionalized phosphonium cations such as triethyl(methoxymethyl)phosphonium ([P_2,2,2_,(1O1)]^+^), [P_2,2,2,5_]^+^, [P_2,2,2_,(2O1)]^+^, and [P_1,1,1,_i_4_]^+^ tend to show lower viscosity and/or higher ionic conductivity than bulkier long‐chain analogues such as tri‐n‐butyl‐*n*‐octylphosphonium ([P_4,4,4,8_]^+^) and [P_6,6,6,14_]^+^, especially when similar anion families are compared [26, 27, 34]. These trends are consistent with reduced molecular volume, weaker dispersive interactions, modified ion organization, and improved segmental mobility in compact or functionalized cation structures [26, 27, 34].

**FIGURE 3:**
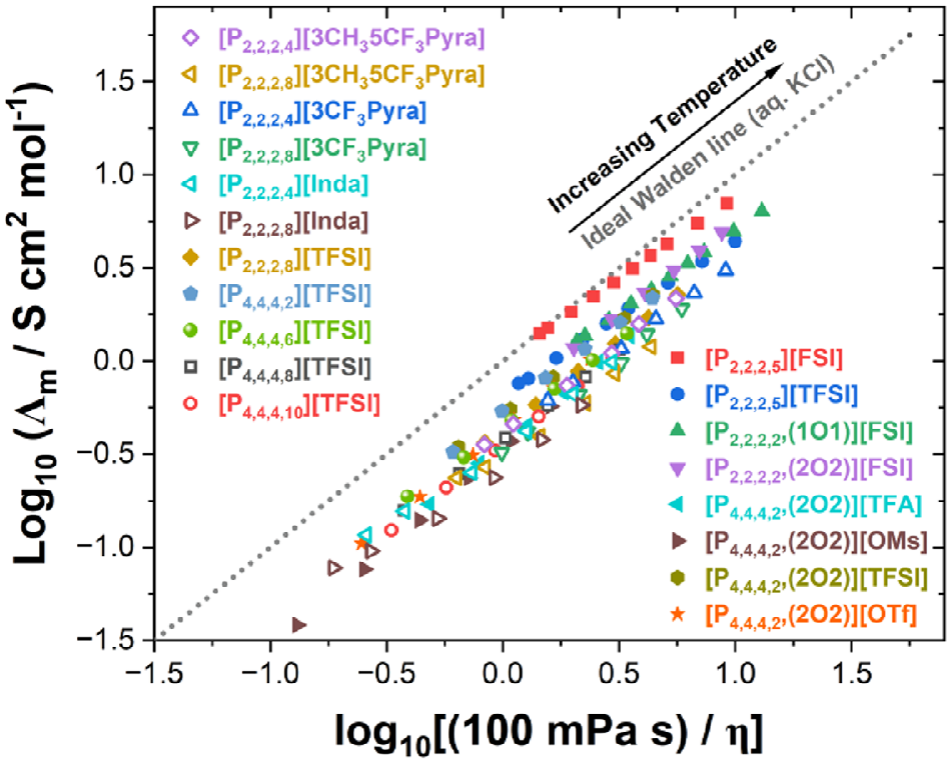
Comparative Walden plot of selected neat phosphonium‐based ILs calculated from literature viscosity, density, and ionic‐conductivity data. The molar conductivity, log_10_(*Λ*_m_/S cm^2^ mol^−1^), is plotted against normalized fluidity, log_10_[(100 mPa s) *η* ^−1^]. The dotted line represents the conventional ideal Walden reference line, commonly used as a qualitative benchmark for fully dissociated dilute electrolytes; it should not be interpreted as an experimentally matched KCl reference for the present neat IL systems. The arrow indicates the direction of increasing temperature. Electrolytes located closer to the reference line show more efficient charge transport relative to viscosity, whereas larger downward deviations indicate stronger correlated ion motion, aggregation, or reduced effective charge mobility. Because the plotted values refer to neat phosphonium ILs, the trends may change substantially after metal‐salt addition, solvent dilution, gel formation, or DES‐based formulation. Abbreviations: [TFA]¯ = trifluoroacetate; [Oms]¯ = methanesulfonate; [Otf]¯ = trifluoromethanesulfonate; [Inda]¯ = indazolide; [Pyra]¯ = pyrazolide. Prefixes indicate ring substitution, for example, 3CF_3_Pyra = 3‐(trifluoromethyl)pyrazolide, 3CH_3_5CF_3_Pyra = 3‐methyl‐5‐(trifluoromethyl)pyrazolide, and 3CF_3_5CF_3_Pyra = 3,5‐bis(trifluoromethyl) pyrazolide. Data were compiled and replotted from Refs. [26, 27, 34].

The compiled data originate from different phosphonium IL families and experimental protocols and should therefore be interpreted as comparative trends in the literature rather than as directly comparable absolute values [26, 34, 112]. Because the plotted values describe neat phosphonium ILs without dissolved metal salts, they should not be interpreted as direct metal‐ion transport numbers or direct descriptors of battery‐electrolyte performance [26, 112, 123]. The addition of Li^+^, Na^+^, Zn^2+^, or other metal salts can alter cation–anion coordination, ion association, viscosity, conductivity, and transference behavior, thereby shifting the electrolyte position in the Walden plot [26, 112, 123]. Studies on phosphonium IL/Li‐salt mixtures have shown that salt addition generally strengthens ion correlations and alters transport characteristics compared with the neat IL [112]. For battery applications, Walden analysis should therefore be interpreted together with salt concentration, transference behavior, interphase chemistry, and electrode compatibility [4, 9, 10, 26, 27, 34, 35, 64, 76, 101, 146].

### 2.5 Interpreting Thermal Stability and Apparent ESW Values

Thermal decomposition behavior is an important safety‐relevant property of phosphonium ILs, but it should be discussed separately from apparent electrochemical stability, because the two are measured by different methods and governed by distinct degradation pathways [14, 27, 68, 69]. In TGA, the apparent *T*_dec_ depends not only on cation and anion structure but also on sample purity, residual halides, water content, metal‐containing impurities, atmosphere, heating rate, pan material, and the criterion used to define decomposition onset [14, 27, 68, 69]. Therefore, reported *T*_dec_ values should be treated as protocol‐dependent descriptors rather than intrinsic constants of a phosphonium cation or anion [14, 27, 68, 69, 150]. As emphasized by Maton et al., *T*_onset_ and *T*_10%_ values obtained from ramped‐temperature TGA can overestimate practical thermal stability because decomposition may occur at lower temperatures under long‐term isothermal conditions [150].

Figure 4 summarizes representative thermal‐decomposition and fire‐hazard examples that illustrate three recurring themes in phosphonium ILs, including thermal–flammability coupling, impurity sensitivity, and cation–anion dependence [14, 69]. The new thermal–flammability safety map in Figure 4A compares representative quaternary phosphonium ILs using *T*_onset_, TTI, and pkHRR [14]. The [P_4,4,4,4_][NTf_2_] system combines the highest *T*_onset_, longest TTI, and lowest pkHRR, whereas bromide‐based phosphonium ILs, especially dodecyltributylphosphonium ([P_4,4,4,12_][Br]), show lower onset temperatures, shorter ignition times, and higher pkHRR [14]. This trend indicates that thermal robustness alone is insufficient for safety ranking; ignition delay and combustion intensity must also be considered [14].

**FIGURE 4:**
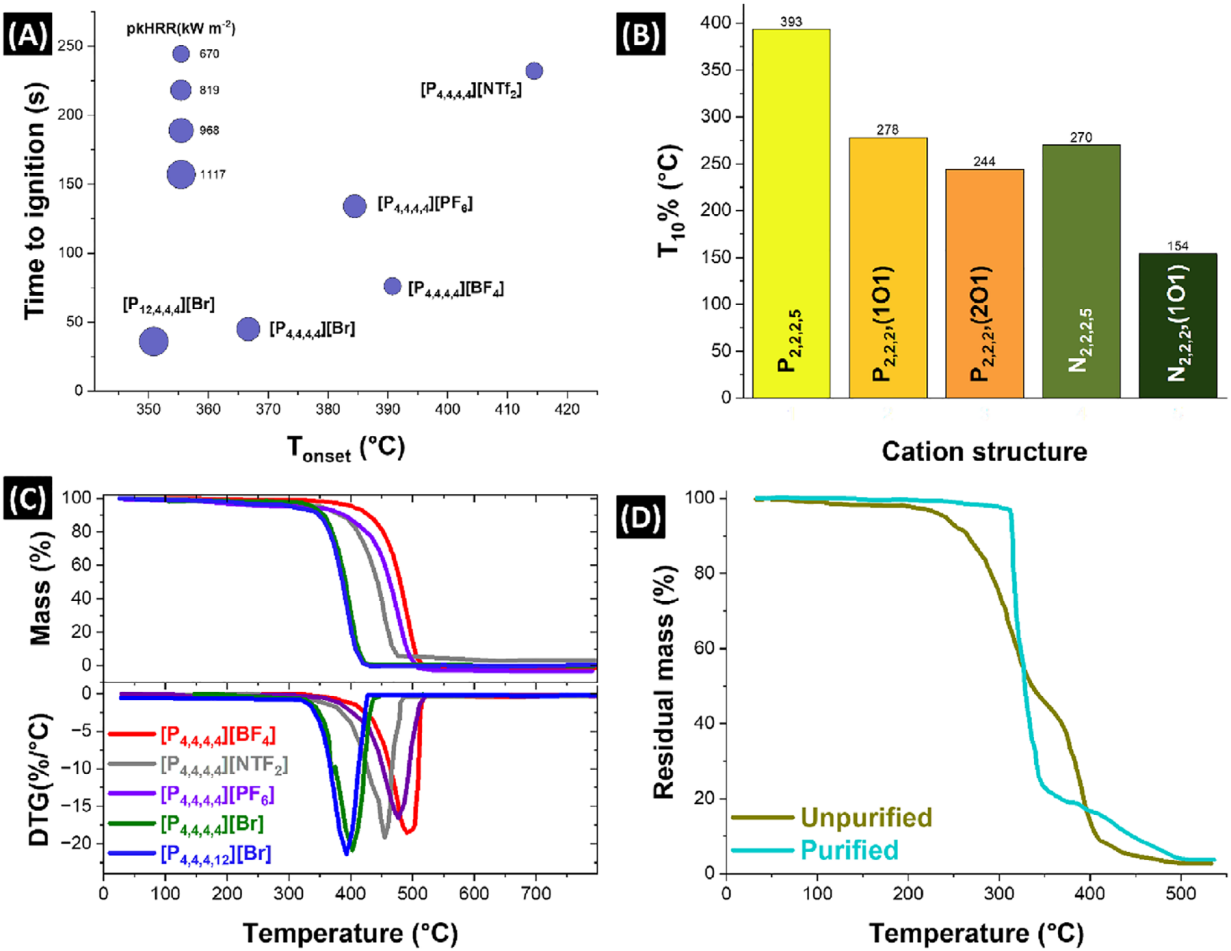
Representative thermal decomposition and flammability behavior of phosphonium salts and quaternary phosphonium ILs. (A) Thermal–flammability safety map of representative quaternary phosphonium ILs. The *x*‐axis represents *T*_onset_, the *y*‐axis represents TTI, and bubble size represents pkHRR. (B) Comparison of *T*_10%_ values for selected phosphonium and ammonium ILs with the common [N(CN)_2_]¯ anion. (C) TG and DTG profiles of representative quaternary phosphonium salts, illustrating the influence of cation and anion structure on thermal‐decomposition behavior. (D) Thermogravimetric comparison of a previously reported impurity‐containing [P_6,6,6,14_][BOB] sample and a high‐purity sample, illustrating the influence of boron‐containing anionic impurities and water on thermal‐decomposition behavior. Data in panel A were replotted from Ref. [14]. Data in panel B were replotted from Ref. [150]. Panel C was adapted from Ref. [14] with permission from Springer Nature Customer Service Centre GmbH. The adapted material is not included under the CC BY license of the present article. Panel D was adapted from Ref. [69] under the CC BY 3.0 license.

THR increased in the order [P_4,4,4,4_][NTf_2_] < [P_4,4,4,4_][PF_6_] < [P_4,4,4,4_][Br] < [P_4,4,4,12_][Br] < [P_4,4,4,4_][BF_4_], further indicating that anion identity and alkyl‐chain structure can both influence fire‐hazard behavior [14]. The comparison in Figure 4D shows that the previously reported impurity‐containing [P_6,6,6,14_][BOB] sample undergoes mass loss at lower temperatures than the high‐purity sample. The original study attributes this difference to boron‐containing transition anionic complexes and associated water, highlighting the importance of synthesis and sample purity when evaluating thermal stability [69]. The comparison of [P_6,6,6,14_][BOB] with Na[BOB] and [P_6,6,6,14_][Cl] further illustrates that cation–anion pairing, anion chemistry, and purity collectively determine thermal‐decomposition behavior [69]. The TG/DTG profiles in Figure 4C further show that anion identity strongly influences the decomposition behavior of salts sharing the [P_4,4,4,4_]^+^ cation [14]. The comparison between [P_4,4,4,4_][Br] and [P_4,4,4,12_][Br] additionally indicates that cation alkyl‐chain architecture can modify the decomposition profile even when the anion is unchanged [14].

Together, these comparisons show that the apparent thermal stability of phosphonium salts reflects the combined effects of cation and anion structure rather than the phosphonium cation alone. These examples should not be interpreted as a complete ranking of the thermal stability of phosphonium ILs [14, 69]. To enable meaningful comparison, thermal data should always be reported together with the relevant experimental conditions (heating rate, atmosphere, sample purity, decomposition criterion, etc.) [14, 27, 68, 69, 150]. This is especially important for borate‐, chloride‐, bromide‐, and fluorinated‐anion systems, where impurities, alkyl‐chain decomposition, and anion‐derived gas‐phase reactions can strongly affect the apparent thermal and combustion profiles [14, 69].

## 3 Interphase Chemistry and Metal/Electrolyte Compatibility

### 3.1 From Bulk Transport to Interphase‐Controlled Performance

Figure 5 summarizes the metal‐specific interfacial roles of phosphonium‐based electrolytes. In metal batteries, phosphonium‐electrolyte performance is governed not only by bulk transport, but also by the chemistry of the metal/electrolyte interface [5, 10, 69, 84]. In practical cells, decomposition products from phosphonium cations, anions, dissolved metal salts, co‐solvents, and trace impurities can collectively form interphases that control metal plating/stripping reversibility, dendrite growth, corrosion, gas evolution, and long‐term cycling stability [10, 23, 84, 99, 151]. Phosphonium ILs influence these interfaces through three coupled mechanisms: modification of local ion association and metal‐ion solvation, changes in desolvation barriers at the electrode surface, and formation of cation‐ or anion‐derived interphase species during electrochemical decomposition [26, 35, 63, 84, 98, 99].

**FIGURE 5:**
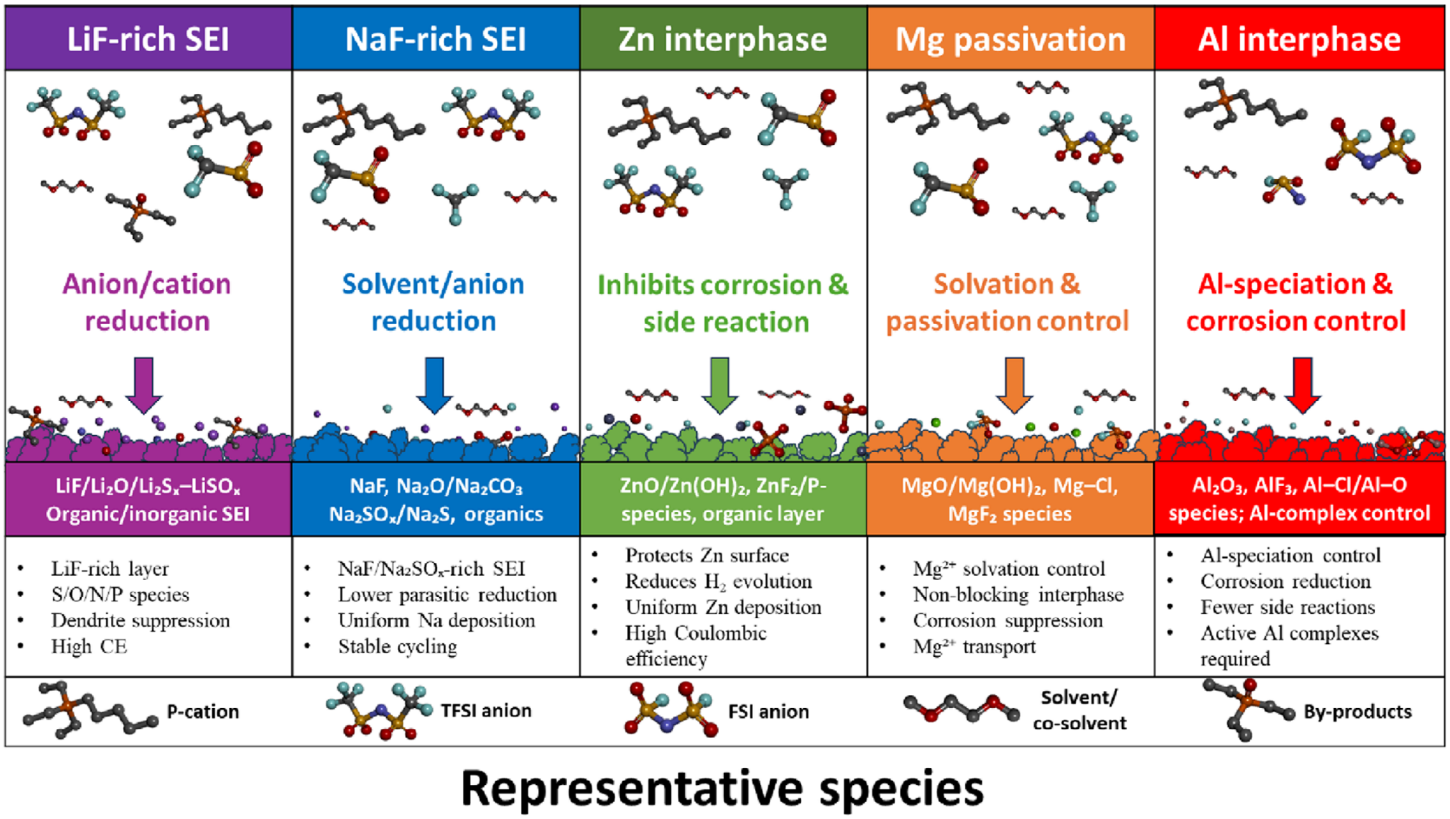
Representative interfacial roles of phosphonium‐based electrolytes in metal‐battery systems. In Li and Na systems, [FSI]^−^/[TFSI]^−^‐containing phosphonium formulations can promote the formation of an inorganic‐rich SEI containing fluoride‐, oxide‐, sulfur‐oxygen‐, and organic decomposition products [5, 10, 76, 84]. In Zn systems, phosphonium‐derived DES or hybrid electrolytes can regulate corrosion, hydrogen evolution, and Zn deposition [23, 151]. In Mg and Al systems, phosphonium electrolytes may modify solvation, passivation, corrosion, and metal‐complex chemistry; however, non‐blocking Mg interphases and active Al complexes remain essential for reversible metal electrochemistry [3, 8, 9, 17, 69, 146]. The schematic was created by the authors as a conceptual summary of literature‐reported interfacial trends; interphase compositions are representative and system‐dependent rather than universal.

The extent of these mechanisms depends strongly on electrode potential, salt concentration, impurity level, and the stability of the specific cation–anion pair [63, 84, 98, 151]. Anions such as [FSI]¯ and [TFSI]¯ can generate inorganic‐rich interphases containing fluoride‐, oxide‐, sulfur–oxygen‐, or sulfonyl‐derived species, while phosphonium‐cation fragments may contribute organic or phosphorus‐containing surface components [10, 63, 84, 98]. These processes provide a mechanistic link between bulk electrolyte structure and interphase formation. A metal ion approaches the electrode within a coordination environment formed by anions, solvent molecules, and other ionic species. Near a polarized electrode, the relative concentrations of phosphonium cations, anions, metal‐containing complexes, and cosolvents can differ markedly from those in the bulk electrolyte [152, 153].

Partial or complete desolvation of the metal ion must then occur before charge transfer and metal deposition [154]. At the same time, electrolyte species present at the interface may undergo potential‐dependent reduction or oxidation, generating the first SEI or CEI components [155, 156]. The resulting interphase subsequently alters ion transfer and further electrolyte decomposition. Phosphonium cations can therefore affect interphase formation indirectly by modifying solvation and interfacial ion organization, or directly when phosphonium‐derived decomposition products become part of the interphase [10, 63, 84, 99].

### 3.2 Metal‐Specific Interfacial Challenges

These system‐dependent interfacial roles are summarized schematically in Figure 5. The dominant interfacial challenge differs across metal chemistries [5, 8, 10, 23, 69, 84]. In Li and Na systems, phosphonium‐based electrolytes are mainly used to stabilize metal plating/stripping, suppress dendrite growth, and limit parasitic electrolyte reduction [10, 76, 84, 99, 157]. However, direct evidence that the resulting interphases are simultaneously ionically conductive, electronically insulating, chemically stable, and mechanically robust remains limited in many reported phosphonium‐electrolyte systems [10, 63, 84]. In Zn systems, interfacial control must additionally suppress hydrogen evolution, corrosion, passivation, and irregular Zn deposition [8, 23, 133, 151].

Recent Zn–Br_2_ work further shows that phosphonium salts can modify cathode‐side halogen chemistry by controlling bromine/polybromide mobility and redox kinetics, emphasizing that phosphonium ions may act as functional interfacial mediators rather than passive electrolyte components [158]. In Mg and Al systems, the key challenge is avoiding blocking interphases while maintaining active transport of Mg^2+^, Al ions, or Al complexes [3, 8, 9, 17]. Therefore, phosphonium‐electrolyte design must be matched to the target metal chemistry, with interphase composition, metal‐ion transport, corrosion suppression, and electrolyte decomposition evaluated together rather than as separate performance metrics [3, 8, 23, 69, 84, 151].

### 3.3 How Phosphonium ILs Affect SEI/CEI

Phosphonium‐based electrolytes affect SEI and CEI formation through coupled decomposition, solvation, and adsorption pathways rather than through a single universal interphase‐forming mechanism [63, 84]. Importantly, the phosphonium cation is not always the main precursor of the interphase. Its role often arises from its ability to modify local ion organization, solvation, and adsorption through differences in size, symmetry, hydrophobicity, and functionalization. Direct phosphonium‐derived SEI or CEI formation occurs only when cation decomposition competes effectively with reduction or oxidation of the anion, salt, solvent, or additive [63, 72, 84, 159]. In Li and Na systems, [FSI]^−^‐containing phosphonium electrolytes can generate inorganic‐rich interphases that contain LiF or NaF, along with sulfur–oxygen and organic decomposition products, thereby suppressing continuous electrolyte consumption and improving metal plating/stripping reversibility [63, 71].

The final SEI composition depends strongly on salt concentration, cation structure, current density, water/halide content, electrode pretreatment, and cycling protocol, meaning that the same phosphonium cation can produce different interfacial outcomes in different electrolyte environments [18, 71]. For Si and graphite/Si anodes, phosphonium [FSI]^−^ additives can stabilize cycling by modifying the SEI during repeated volume expansion and reducing continuous Li/electrolyte consumption [84]. For example, a 10 wt.% [P_1,1,1,_i_4_][FSI] formed a LiF/poly(VC)/P–C‐containing SEI instead of a more Li_2_CO_3_‐rich, high‐impedance interphase, extending Gr/Si cycling lifetime from 107 to 350 cycles [37]. In some [P_1,1,1,_i_4_][FSI]/LiFSI systems, the dominant SEI products are mainly assigned to [FSI]¯ reduction, including LiF, Li_2_O, Li_2_S, LiSO_2_, and Li_2_NSO_2_F, whereas little phosphonium‐cation decomposition is detected [63, 84].

In contrast, the fluorine‐free [P_1,1,1,_i_4_][TCM] /lithium dicyanamide ([LiDCA]) electrolyte forms a bilayer SEI containing [TCM]¯, [DCA]¯, and phosphonium‐cation‐derived species, showing that cation decomposition can contribute when the anion chemistry and reduction sequence favor it [63, 84]. This contrast shows that phosphonium‐cation participation in SEI formation is not an intrinsic feature of the cation class. Instead, it depends on competition among cation, anion, salt, and solvent decomposition pathways [160]. In [FSI]¯‐rich electrolytes, preferential anion reduction can dominate and generate LiF‐ and sulfur‐containing inorganic species, whereas fluorine‐free formulations may shift the decomposition sequence sufficiently for phosphonium‐derived species to become detectable [63, 72, 159]. In sodium systems, phosphonium ILs influence both Na^+^ transport and Na‐metal interphase formation through cation‐dependent solvation, diffusion, and interfacial adsorption [18, 71].

Increasing the fraction of [P_1,1,1_,i_4_][FSI] in mixed [P_1,1,1_,i_4_][FSI]/N‐methyl‐N‐propylpyrrolidinium bis(fluorosulfonyl)imide ([C_3_mpyr][FSI])/sodium bis(fluorosulfonyl)imide (NaFSI) electrolytes increased ionic conductivity and ion‐diffusion coefficients and shifted sodium plating behavior [18, 71]. However, Na‐metal stability also remained strongly dependent on salt concentration and formation protocol. Therefore, phosphonium cations should be considered active regulators of Na^+^ solvation and SEI formation, rather than merely conductivity modifiers [18, 71]. For cathodes, the evidence for phosphonium‐derived CEI formation is still less direct than for Li, Si, Gr/Si, and Na‐metal interfaces [161, 162]. High‐voltage electrolyte studies show that P‐, F‐, N‐, B‐, O‐, and sulfur‐containing decomposition products can form protective CEI/SEI layers that reduce parasitic reactions, transition‐metal dissolution, impedance growth, and gas evolution, but these findings should be separated from direct proof of phosphonium‐derived CEI chemistry [162, 163].

Therefore, improved high‐voltage cycling in phosphonium‐containing formulations should be assigned to phosphonium‐derived CEI only when supported by surface‐sensitive evidence [84, 162]. Overall, phosphonium ILs influence SEI/CEI formation through coupled anion decomposition, cation‐dependent solvation and adsorption, salt‐concentration effects, and electrode‐specific decomposition pathways [18, 63]. The strongest direct evidence currently exists for Li‐metal, Si, graphite/Si, and Na‐metal interfaces, whereas direct CEI evidence for high‐voltage cathodes remains less established and requires more operando and surface‐sensitive characterization [71, 84].

### 3.4 Dynamic Interphase Evolution Under Operating Conditions

Most studies of phosphonium‐based electrolytes characterize the SEI or CEI after cycling, providing a largely static picture of an inherently dynamic interface [37, 160, 164]. During operation, however, interphase composition and morphology can evolve continuously through electrolyte decomposition, dissolution and redeposition of interphase species, mechanical disruption, and reactions at newly exposed electrode surfaces [164]. These processes may be particularly important for Li and Na metal, where repeated plating/stripping continuously changes the electrode surface, and for Si‐containing anodes, where large volume changes can fracture the existing SEI and expose fresh material [164, 165]. Consequently, an interphase identified after cycling may not represent the transient species that controlled charge transfer or degradation during earlier stages of operation.

For phosphonium electrolytes, this dynamic behavior should also be considered in relation to cation–anion competition at the polarized interface. Changes in electrode potential are expected to alter the local enrichment of phosphonium cations, anions, metal‐ion complexes, and solvent molecules, thereby changing which species are preferentially reduced or oxidized [63, 84, 152, 166, 167]. An initially anion‐derived inorganic interphase could therefore evolve as cycling proceeds, while cation‐ or solvent‐derived products may contribute at later stages depending on potential, salt concentration, and electrode chemistry [63, 164, 168]. Whether phosphonium‐derived interphases undergo effective self‐repair after mechanical or chemical disruption remains largely unresolved. Interphase evolution is also spatially heterogeneous. Local variations in current density, electrode morphology, surface defects, and electrolyte composition can produce regions with different interphase thicknesses, compositions, and transport properties [169].

Such heterogeneity is difficult to capture by conventional surface‐averaged measurements and may be particularly important for metal anodes, where non‐uniform ion flux can promote localized deposition and repeated interphase rupture [164]. Three‐dimensional ToF‐SIMS depth profiling can map the lateral and depth‐dependent distribution of inorganic and organic interphase species, while cryogenic electron tomography can preserve beam‐sensitive interfaces and reveal their three‐dimensional morphology at the nanoscale [170, 171, 172]. Combining these approaches with electrochemical measurements would help determine whether phosphonium‐derived species form a spatially uniform protective layer or instead accumulate selectively at defects, high‐current‐density regions, or newly exposed electrode surfaces. Resolving these processes requires characterization that follows the interface during operation rather than relying only on post‐mortem analysis.

Operando or quasi‐operando X‐ray photoelectron spectroscopy (XPS) can track changes in surface composition and oxidation state, electrochemical quartz crystal microbalance with dissipation monitoring (EQCM‐D) can resolve potential‐dependent mass uptake and viscoelastic changes, and electrochemical atomic force microscopy (EC‐AFM) can follow interphase growth and morphological evolution [168, 173, 174, 175]. Complementary operando Raman spectroscopy and EIS can further correlate changes in interfacial chemistry with the evolution of interfacial resistance and charge‐transfer behavior [168, 176]. Applying these methods to systematically varied phosphonium cations and anions would help distinguish transient interfacial adsorption from irreversible decomposition and identify whether particular molecular architectures promote persistent, self‐limiting, or self‐healing interphases.

Another unresolved aspect is competitive adsorption at the polarized electrode surface. In phosphonium electrolytes, the interfacial composition is determined by competition among phosphonium cations, anions, metal‐ion complexes, solvent molecules, and additives rather than by the bulk electrolyte composition alone [177, 178]. As the electrode potential changes, counterions can become enriched in the inner interfacial region while co‐ions are displaced or reoriented, producing potential‐dependent changes in ion packing and local coordination [152, 177, 179]. In metal‐salt‐containing electrolytes, this competition may additionally determine whether a metal‐ion complex can approach the electrode, undergo desolvation, and participate in charge transfer before nearby electrolyte species decompose [154].

Consequently, cation size, functionalization, anion coordination strength, and salt concentration may affect interphase chemistry through competitive adsorption even when the phosphonium cation itself is not electrochemically decomposed. Direct evidence for such competition remains limited in phosphonium battery electrolytes, making potential‐resolved interfacial measurements particularly important. Electrochemical SERS can probe potential‐dependent adsorption, orientation, and replacement of interfacial ions, while EQCM or EQCM‐D can quantify accompanying changes in interfacial mass [168, 173, 177]. Combining these methods with EIS would help distinguish reversible adsorption and ion exchange from irreversible electrolyte decomposition and interphase growth.

### 3.5 Operando/In Situ Infrared Spectroscopy of Interfacial Dynamics

Operando/in situ electrochemical infrared (IR) spectroscopy can complement post‐cycling SEI/CEI analysis by following changes in the electrolyte configuration at polarized interfaces [180, 181]. Attenuated total reflection Fourier‐transform infrared spectroscopy (ATR‐FTIR) can distinguish bands associated with free and metal‐ion‐coordinated solvents and anions, providing potential‐ and time‐dependent information on solvation and ion pairing [180, 181]. Surface‐enhanced infrared absorption spectroscopy (SEIRAS) increases sensitivity to species close to suitably prepared enhancing electrodes, including adsorbates and nascent interphase components [181]. Polarization‐modulation infrared reflection–absorption spectroscopy (PM‐IRRAS) provides complementary information on adsorption and molecular orientation through polarization‐dependent signals and surface‐selection rules, as demonstrated at a glassy‐carbon/deep‐eutectic‐solvent interface [182].

Recent lithium‐battery studies illustrate why these measurements are valuable. In lithium hexafluorophosphate (LiPF_6_)/carbonate electrolytes, Yang et al. combined ATR‐FTIR and SEIRAS to link anion depletion and solvent enrichment during Li plating with subsequent SEI formation. Increasing the temperature from 25°C to 55°C intensified this interfacial restructuring and promoted a more organic‐rich, less stable SEI [181]. At lithium cobalt oxide (LiCoO_2_) cathodes, Luo et al. used ATR‐FTIR and molecular dynamics simulations to resolve nonmonotonic changes in interfacial Li^+^‐associated species during charging, arising from competition between field‐driven migration and Li^+^ released by cathode delithiation [180]. These studies provide methodological examples for investigating phosphonium electrolytes rather than direct evidence for phosphonium‐specific interfacial mechanisms.

Applied to phosphonium formulations, potential‐step and cycling experiments could follow independently assigned bands of phosphonium cations, anions, solvents, and metal‐ion complexes to test how adsorption, desorption, reorientation, and solvation‐shell changes accompany charge transfer. Tracking the appearance, persistence, and disappearance of product bands could also help establish decomposition sequences and identify IR‐active intermediates whose lifetimes exceed the measurement time resolution. Spectral changes that reverse when the potential is returned to its initial value would be consistent with reversible adsorption, coordination, or ion exchange. Persistent product bands, assessed together with interfacial mass and impedance changes, would instead support irreversible interphase formation. However, slow desorption and interphase dissolution must also be considered when interpreting reversibility [180, 181, 182].

Interpretation requires care because bulk‐electrolyte absorption, overlapping bands, surface‐selection rules, detection sensitivity, and background subtraction can obscure interfacial changes [180, 181, 182]. Band intensities may reflect molecular orientation or changes in optical coupling rather than concentration alone [181, 182]. Conventional ATR‐FTIR configurations may provide weak signals from solid interphase products relative to the liquid electrolyte [180]. For phosphonium systems, reliable interpretation is aided by validated band assignments, clearly specified reference potentials and temperatures, stable optical geometry, and controlled water and oxygen levels. Comparison with bulk spectra and complementary EIS, EQCM‐D, and surface analysis would help distinguish genuine changes in adsorption and decomposition from optical artifacts or impurity‐related reactions.

### 3.6 Desolvation and Interfacial Charge‐Transfer Kinetics

Interfacial kinetics depend not only on bulk ionic conductivity but also on the energy required to reorganize or partially remove the metal‐ion solvation shell before charge transfer. This distinction becomes particularly important for multivalent ions such as Zn^2+^ and Mg^2+^ because their higher charge density produces stronger ion–solvent and ion–anion interactions than those typically encountered for Li^+^ or Na^+^ [154, 183]. Consequently, an electrolyte with relatively high bulk conductivity may still exhibit slow metal deposition if desolvation or transport through the interphase is kinetically unfavorable [154, 183]. For Zn^2+^, the interfacial kinetic barrier includes coupled solvent removal, ion transfer through the interphase, and electron transfer at the metal surface [183]. Temperature‐dependent EIS provides a useful way to compare these processes because the apparent activation energy derived from the charge‐transfer resistance reflects the overall interfacial kinetic barrier [154, 183].

For example, electrolyte and interphase engineering in aqueous Zn systems has been shown to decrease apparent Zn^2+^ desolvation/charge‐transfer activation energies from approximately 70 to 55 kJ mol^−1^ [183], while other engineered interfaces have reduced these values to below 30 kJ mol^−1^ [184]. These results illustrate that changes in the interfacial coordination environment can strongly affect Zn deposition kinetics even when bulk conductivity changes are comparatively modest. This principle is directly relevant to phosphonium‐containing Zn electrolytes. Ether‐functionalized phosphonium cations have been shown to restructure the Zn/electrolyte interface and improve Zn plating/stripping reversibility, indicating that phosphonium functionalization can influence interfacial coordination and decomposition rather than acting only as an inert supporting ion [185].

However, quantitative temperature‐dependent EIS or activation‐energy analysis has rarely been reported specifically for phosphonium–Zn systems. Such measurements would be valuable for separating improvements in bulk transport from genuine reductions in Zn^2+^ desolvation and charge‐transfer barriers. The kinetic challenge is even greater for Mg^2+^ because its strong coordination produces slower ligand exchange and larger desolvation penalties [154]. Mg electrodeposition can therefore involve sequential solvent or ligand removal, reorganization of Mg‐containing complexes, electron transfer, and transport through any surface film formed during electrolyte decomposition [154]. Temperature‐dependent EIS, together with spectroscopic identification of Mg coordination environments, can help distinguish bulk‐ion transport from interfacial resistance [154, 186]. Recent Mg‐electrolyte studies show that modifying the solvation structure can substantially reduce interfacial resistance and improve plating/stripping kinetics, whereas poorly passivated interfaces can develop markedly higher resistance during cycling [186, 187]. For phosphonium electrolytes, comparable quantitative studies remain scarce, highlighting the need to correlate cation architecture and anion chemistry with Mg^2+^ coordination, activation energy, interfacial resistance, and reversible deposition.

### 3.7 Characterization Needs

Future studies could benefit from complementing electrochemical testing with selected surface‐sensitive and operando methods, chosen according to the specific interfacial question and available instrumentation. Relevant approaches include XPS, time‐of‐flight secondary ion mass spectrometry (ToF‐SIMS), cryogenic transmission electron microscopy (cryo‐TEM), Raman spectroscopy, operando/in situ infrared spectroscopy (ATR‐FTIR, SEIRAS, and PM‐IRRAS), electrochemical quartz crystal microbalance (EQCM), and gas analysis [63, 84, 98, 146, 180, 181, 182]. Used individually or in complementary combinations, these techniques can help distinguish genuine interphase stabilization from apparent stability caused by kinetic limitations or suppressed side reactions [10, 23].

Collectively, phosphonium ILs are relevant not only as low‐volatility, thermally robust bulk electrolytes but also as interfacial modifiers that regulate solvation, desolvation, decomposition pathways, corrosion, gas evolution, and metal deposition [84, 99]. This interfacial perspective provides the mechanistic bridge between the structure–property relationships discussed above and the system‐specific applications discussed below [3, 8, 10, 23, 69, 84].

## 4 Application Relevance in Metal Batteries

A system‐level overview of representative phosphonium‐based electrolyte applications, demonstrated roles, and remaining limitations is provided in Table 2. Phosphonium‐based electrolytes have been investigated across lithium, sodium, zinc, magnesium, and aluminum batteries, as well as supercapacitors, hybrid gels, polymeric ILs, and deep‐eutectic‐type formulations [3, 4, 8, 188]. These combined properties make phosphonium‐based electrolytes relevant in applications where electrolyte safety, interfacial stability, metal compatibility, or voltage stability limits conventional systems [14, 21, 75, 98]. Transport, stability, cell configuration, and cycling data are summarized in Table S2.

**TABLE 2:** Representative application roles and major limitations of phosphonium‐based electrolytes across metal batteries, supercapacitors, and quasi‐solid electrolyte systems. Entries summarize reported functions at the system level and should be interpreted as qualitative application maps rather than universal performance rankings. Detailed conductivity, viscosity, apparent ESW, cell configuration, and cycling data are provided in Table S2.

System	Representative phosphonium‐based electrolyte	Main demonstrated role	Main limitation
Li batteries [15, 63, 75, 84, 98]	[FSI]¯/[TFSI]¯ ILs; carbonate–IL hybrids; phosphonium additives	High‐voltage safety; Li/Si interphase control; reduced flammability	High viscosity; wetting limits; fluorinated‐anion concerns
Na batteries [10, 71, 76, 99]	[P_1,1,1,_i_4_][FSI]–NaFSI; amino‐phosphonium FSI electrolytes; mixed FSI ILs	Na plating/stripping; Na‐ion full cells; thermal safety	Limited cathode diversity; cost/viscosity at high salt loading
Zn systems [23, 151, 185, 189]	Phosphonium–TFSI salts; phosphonium DESs	Zn interphase regulation; dendrite suppression; Zn–air electrolytes	High DES viscosity; limited long‐term validation
Mg systems [8, 9, 17]	[P_6,6,6,14_][Cl]‐based Mg electrolytes; Mg‐relevant phosphonium ILs	Mg/electrolyte interface control; metal–air compatibility	Mg^2+^ transport, passivation, and corrosion
Al systems [3, 8, 69, 146]	Borate‐, [TFSI]^−^‐, or chloride‐containing phosphonium ILs	Thermal stability; corrosion control; Al‐speciation design	Reversible Al plating requires active Al complexes
Supercapacitor [94, 100, 144, 190]	[P_6,6,6,14_]‐based ILs; nitrile electrolytes; PEDOT composites	High‐voltage, low‐volatility capacitance enhancement	Large ion size; limited high‐rate performance
Hybrid gel/DES [22, 24, 144, 191]	Phosphonium IL–polymer gels; polymeric ILs; phosphonium DESs	Leakage resistance; mechanical stability; quasi‐solid design	Lower conductivity; composition/water/ impurity sensitivity

The role of phosphonium ILs varies by system; for example, in Li and Na batteries, the main issues are metal/electrolyte interphases, metal plating/stripping, and high‐voltage stability [14, 27, 69]. In Zn, Mg, and Al systems, they more often serve as interfacial‐control or speciation‐control components, where corrosion suppression, hydrogen evolution, passivation, and active metal‐complex transport become decisive [35, 69, 84, 146]. In supercapacitors, polymer gels, and DES‐type formulations, the main value lies in safety enhancement, leakage resistance, wider voltage operation, and quasi‐solid‐electrolyte design [3, 4, 8, 23, 69, 89]. The following subsections therefore emphasize demonstrated system‐specific functions, practical limitations, and validation gaps rather than ranking phosphonium ILs by generic bulk properties.

### 4.1 Lithium Systems

Lithium batteries remain the most established platform for evaluating phosphonium‐based IL electrolytes because their safety, oxidative stability, ion‐transport behavior, and interfacial chemistry can be directly compared with those of conventional carbonate electrolytes [4, 5, 15, 28]. Phosphonium ILs paired with [FSI]¯ or [TFSI]¯ anions have been investigated as neat electrolytes, highly concentrated salt‐containing electrolytes, hybrid carbonate/IL electrolytes, polymer‐ or gel‐based electrolytes, and electrolyte additives [37, 75, 192, 193]. These systems have been applied in lithium‐metal, anode‐free, high‐voltage cathode, graphite/silicon, and silicon‐anode cells [30, 72, 135, 194, 195]. Figure 6 compares the cycling performance of LFP‖Si/Gr full cells using carbonate and phosphonium‐containing hybrid electrolytes, highlighting the strong dependence of performance on LiFSI concentration [72]. In high‐voltage half‐cells, phosphonium‐containing hybrid electrolytes improved the performance of LiMn_2_O_4_ǁLi and LiNi_0.8_Co_0.15_Al_0.05_O_2_ǁLi cells [15, 16].

**FIGURE 6:**
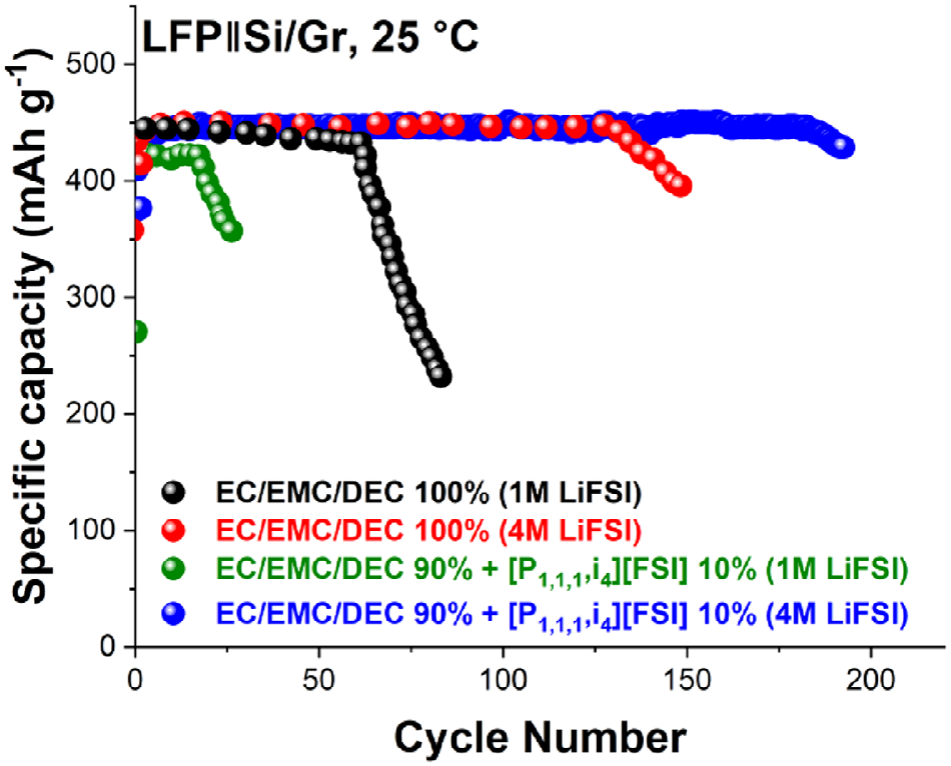
Redrawn cycling comparison of LFPǁSi/Gr full cells using carbonate and phosphonium‐containing hybrid electrolytes. The electrolyte containing 10 wt.% [P_1,1,1,_i_4_][FSI] with 4 m LiFSI shows the longest cycling stability, whereas the same phosphonium additive at 1 m LiFSI is less effective. The comparison highlights the strong dependence on LiFSI concentration. Data redrawn from Ref. [72].

These improvements were attributed to reduced flammability, lower interfacial resistance, and suppressed parasitic reactions, resulting in improved capacity retention and Coulombic efficiency at elevated voltages and temperatures [15, 16]. In lithium‐metal and anode‐free configurations, super concentrated phosphonium [FSI]¯ electrolytes have shown high Li plating/stripping Coulombic efficiency, improved Li morphology, enhanced water tolerance, and compatibility with high‐areal‐capacity cathodes [70, 135, 159]. In [FSI]^−^‐based systems, SEI formation on Li metal and Si‐containing anodes is strongly influenced by Li‐salt concentration, current density, cation structure, electrode preconditioning, and electrolyte composition, often resulting in inorganic‐rich interphases containing LiF and sulfur–oxygen species [72, 135, 195, 196].

For silicon and Gr/Si anodes, phosphonium [FSI]¯ electrolytes or additives can improve cycling by stabilizing the SEI and reducing continuous electrolyte consumption [159, 160, 196]. Beyond molecular electrolytes, polymerizable and polymer‐supported phosphonium systems further expand the design space by improving safety, mechanical robustness, and interfacial stability [63]. Meanwhile, phosphonium IL‐based solid‐ and gel‐polymer electrolytes and organic ionic plastic crystal/poly(vinylidene fluoride) (OIPC/PVDF) composites have been developed to enhance safety, mechanical robustness, and interfacial stability with Li [22, 24, 63, 65, 98]. Despite these advantages, practical lithium applications remain limited by viscosity, Li^+^ transport, electrode wetting, separator compatibility, cost, and sustainability concerns related to fluorinated anions [4, 30, 63, 64, 195, 197].

Therefore, practical LIB strategies favor small‐cation [FSI]^−^ electrolytes, super concentrated LiFSI/phosphonium systems, hybrid carbonate/IL formulations, fluorine‐free phosphonium concepts, polymerizable additives, and gel‐ or solid‐state phosphonium electrolytes [4, 15, 30, 63, 64, 72, 98, 135, 194, 195, 197]. A recent high‐concentration hybrid‐electrolyte study showed that adding 10 wt.% [P_1,1,1,_i_4_][FSI] to EC/EMC/DEC did not improve Si/Gr cycling at 1 m LiFSI, whereas the same additive with 4 m LiFSI produced the longest LiFePO_4_(LFP)ǁSi/Gr cycling stability [72]. This improvement was attributed to a LiF‐rich, phosphorus‐containing SEI that suppressed continuous side reactions [72]. Thus, phosphonium additives should be evaluated as formulation‐dependent interphase modifiers rather than universal performance enhancers [72].

### 4.2 Sodium Systems

SIBs are among the most mature post‐lithium energy‐storage technologies because sodium is abundant, low‐cost, and compatible with many manufacturing concepts already established for LIBs [7, 198, 199]. In addition, Na metal offers a high theoretical gravimetric capacity of ≈1166 mAh g^−1^ and a volumetric capacity of ≈1128 mAh cm^−3^, making it an attractive anode for next‐generation high‐energy systems [6, 7, 8, 9, 10, 71, 76, 99]. Despite these advantages, sodium electrochemistry differs substantially from that of lithium owing to the larger ionic radius of Na^+^, distinct solvation structures, and more challenging interfacial chemistry, which often result in unstable Na deposition, low Coulombic efficiency, and accelerated electrolyte decomposition [6, 7, 8, 9, 10, 71, 76, 99]. For phosphonium‐based sodium electrolytes, the most relevant advantages are high NaFSI solubility, useful ionic conductivity in compact‐cation [FSI]¯ systems, broad operating‐temperature windows, and the ability to regulate Na‐metal interfacial chemistry [71, 76, 99].

In particular, compact phosphonium cations such as [P_1,1,1_,i_4_]^+^ and Tris(amino)phosphonium ([TAP]^+^) cations paired with [FSI]¯ or [TFSI]¯ anions provide useful electrolyte properties for sodium‐metal and selected SIBs [71, 76, 99]. Phosphonium‐based [FSI]^−^ electrolytes tailor Na^+^ solvation and interfacial chemistry, promoting stable Na plating/stripping while suppressing side reactions [10, 71]. For example, near‐saturated [P_1,1,1_,i_4_][FSI]‐NaFSI electrolytes showed superior Na‐metal cycling compared with pyrrolidinium analogues, supporting areal capacities up to 4 mAh cm^−2^, whereas the corresponding [C_3_mpyr][FSI] system maintained stable cycling only up to 1 mAh cm^−2^ [10]. Surface analysis further showed that controlled water addition promoted the formation of a NaF‐rich interphase and improved the stability of Na metal [10]. Cation chemistry also strongly affects sodium electrochemistry [71].

Mixed‐cation IL electrolytes based on [P_1,1,1_,i_4_][FSI], [C_3_mpyr][FSI], and NaFSI showed that increasing the [P_1,1,1_,i_4_][FSI] fraction slightly lowered the glass‐transition temperature, increased ionic conductivity and cation/anion diffusion coefficients, and promoted earlier Na plating with higher current density [71]. Beyond sodium‐metal systems, phosphonium‐based electrolytes have also been evaluated in sodium‐ion full cells [76, 99]. A saturated NaFSI/[P_1,1,1_,i_4_][FSI] electrolyte was successfully coupled with P2‐ and O3‐type Na_2/3_[Fe_2/3_Mn_1/3_]O_2_ cathodes and outperformed conventional organic electrolytes in capacity retention and long‐term cycling at 50°C [76]. [TAP][FSI] electrolytes containing 50 mol% NaFSI also supported Na plating/stripping and NaǁNaFePO_4_ full‐cell operation with high capacity retention and Coulombic efficiency close to 99.5% [71, 76, 99].

These studies demonstrate that phosphonium electrolytes stabilize Na‐metal interfaces and enable selected Na‐ion full cells when the cation structure, [FSI]^−^ concentration, and operating temperature are appropriately matched [71, 76, 99]. However, beneficial behavior in Na‐metal and Na‐ion cells should not be generalized to all SIB chemistries [71, 76, 99, 200]. In Na–O_2_ hybrid electrolytes, discharge‐product morphology, NaO_2_ purity, side‐product formation, and long‐term cyclability depend strongly on IL cation/anion chemistry and Na^+^/superoxide speciation [200]. More recent phosphonium–[FSI]^−^ formulations, however, have demonstrated substantially improved Na–O_2_ cycling after optimization of cation chemistry, anion identity, and Na‐salt concentration, indicating that performance is strongly dependent on electrolyte speciation and formulation [33]. A direct comparison between phosphonium‐ and pyrrolidinium‐based [FSI]^−^ electrolytes shows that bulk transport properties are important but insufficient to predict Na‐metal performance in Figure 7.

**FIGURE 7:**
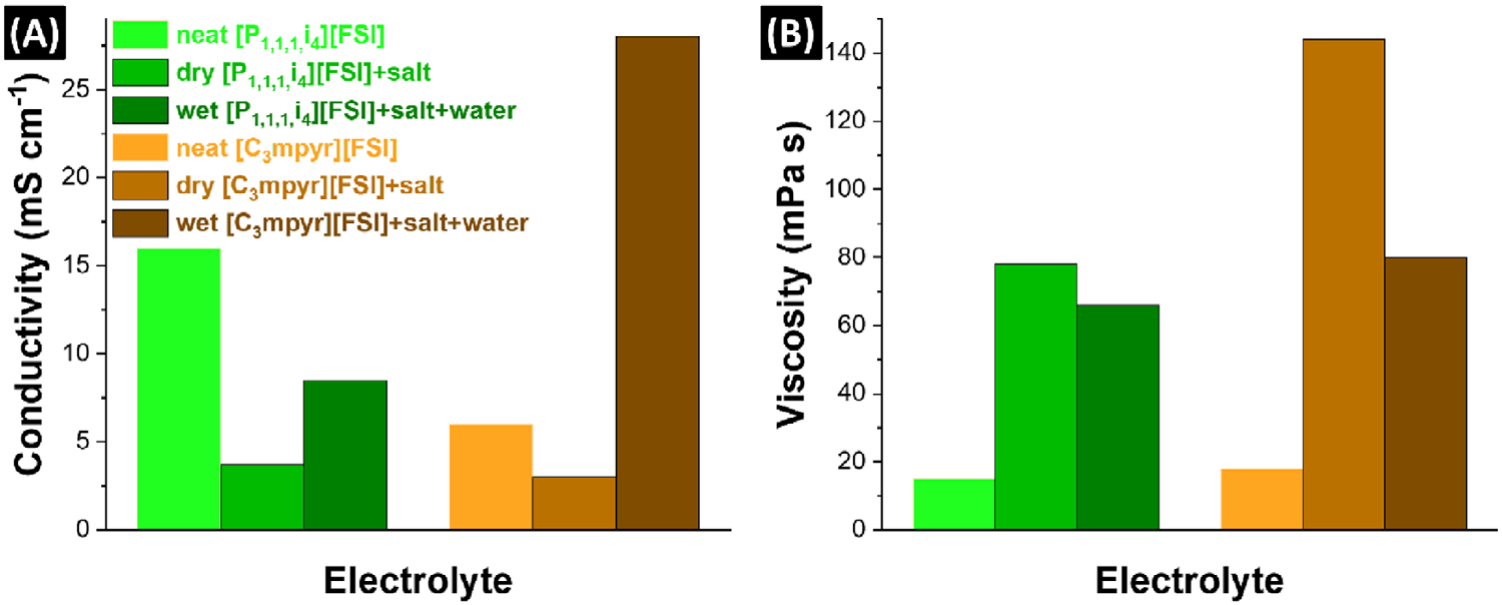
Bulk transport comparison of phosphonium‐ and pyrrolidinium‐based FSI electrolytes for sodium‐metal batteries. Conductivity and viscosity values are shown for neat ILs and super concentrated NaFSI‐containing electrolytes in dry and wet states at 50°C. The [P_1,1,1,_i_4_][FSI]/NaFSI electrolyte shows lower viscosity than [C_3_mpyr][FSI]/NaFSI in the dry salt‐containing state, whereas water addition strongly increases conductivity, especially for [C_3_mpyr][FSI]/NaFSI. These data illustrate that conductivity and viscosity alone do not fully determine Na‐metal cycling behavior; interfacial chemistry and SEI formation must also be considered. Data redrawn from Ref. [10].

In super concentrated NaFSI electrolytes, [P_1,1,1,_i_4_][FSI] exhibits lower viscosity than [C_3_mpyr][FSI] in the dry salt‐containing state, while the addition of ≈1000 ppm water increases ionic conductivity in both systems [10]. However, the very high conductivity of wet [C_3_mpyr][FSI]/NaFSI does not translate into superior high‐capacity Na‐metal cycling, whereas [P_1,1,1,_i_4_][FSI]/NaFSI supports more demanding Na plating/stripping [10]. This comparison indicates that phosphonium cation chemistry affects Na‐metal reversibility through both bulk transport and interfacial processes, including SEI composition and deposition morphology. In Na–O_2_ systems, excessive stabilization of superoxide intermediates by the phosphonium electrolyte led to rapid cell failure, illustrating that favorable Na‐metal behavior does not necessarily translate into improved oxygen‐electrode performance [200].

Overall, phosphonium‐based electrolytes represent a promising platform for Na‐metal batteries and selected Na‐ion chemistries, particularly where high NaFSI solubility, thermal stability, interfacial control, and [FSI]^−^‐based formulations are required [71, 76, 99, 200]. Direct demonstrations with Prussian‐blue analogue (PBA) cathodes remain sparse in the phosphonium‐electrolyte literature; therefore, PBAǁhard‐carbon or PBAǁNa‐metal cells should be framed as a promising future direction rather than an established phosphonium‐electrolyte platform [71, 76, 99]. Representative phosphonium‐based sodium electrolyte formulations and performance metrics are summarized in Table 2 and Table S2 [71, 76, 99].

### 4.3 Zinc Systems

ZIBs are attractive for safe, low‐cost energy storage because Zn is inexpensive, comparatively safe, and naturally abundant, and it offers high theoretical gravimetric and volumetric capacities of ≈820 mAh g^−1^ and ≈5854 mAh cm^−3^, respectively [23, 151, 185]. However, practical Zn‐metal batteries are limited by hydrogen evolution, Zn corrosion, surface passivation, shape change, and dendritic Zn growth, especially in aqueous electrolytes where water activity strongly affects anode stability [151, 185]. Therefore, phosphonium‐based Zn electrolytes are most relevant when they regulate Zn^2+^ solvation, water activity, interfacial charge transfer, Zn deposition morphology, corrosion behavior, and cathode compatibility without introducing excessive viscosity or transport limitations [23, 35, 151, 185, 189]. The most direct evidence for phosphonium chemistry in rechargeable Zn‐metal batteries comes from functionalized phosphonium‐TFSI supporting salts in aqueous Zn(TFSI)_2_ electrolytes [185].

Tributyl(2‐methoxyethyl)phosphonium ([P_4,4,4_,(2O1)]^+^) provides stronger water‐screening capability than tributylmethylphosphonium ([P_4,4,4,1_]^+^) and promotes interphase chemistry that enables highly reversible Zn plating/stripping [185]. As summarized in Figure 8, the average Zn plating/stripping CE increases from 78.1% in 4 m Zn(TFSI)_2_ to 92.09% with 4 m [P_4,4,4,1_][TFSI] and 98.10% with 4 m [P_4,4,4_,(2O1)][TFSI] [185]. The ether‐functionalized electrolyte further supports dendrite‐free ZnǁZn cycling for >6400 h at 0.5 mA cm^−2^/0.5 mAh cm^−2^ and remains stable for >280 h under more aggressive conditions of 2.5 mA cm^−2^/2.5 mAh cm^−2^ [185]. Full‐cell testing with a Na_2_V_6_O_16_·1.63H_2_O cathode also demonstrated 2000 cycles at 300 mA g^−1^, showing that phosphonium‐cation functionality translates Zn‐metal reversibility into practical cell operation [185].

**FIGURE 8:**
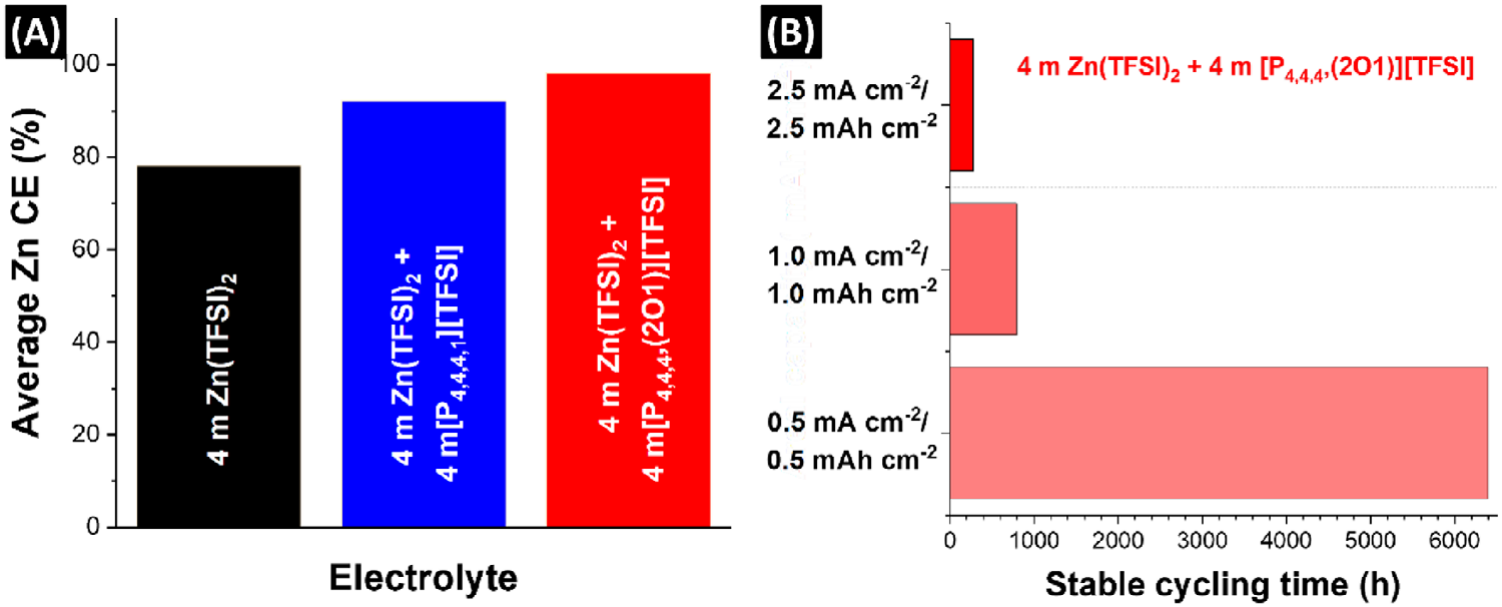
Effect of phosphonium supporting‐salt cation functionality on Zn‐metal reversibility in aqueous 4 m Zn(TFSI)_2_ electrolytes. (A) Average Zn plating/stripping Coulombic efficiency for 4 m Zn(TFSI)_2_, 4 m Zn(TFSI)_2_ + 4 m [P_4,4,4,1_][TFSI], and 4 m Zn(TFSI)_2_ + 4 m [P_4,4,4_,(2O1)][TFSI]. The ether‐functionalized phosphonium salt gives the highest average Zn CE. (B) Stable ZnǁZn cycling time for 4 m Zn(TFSI)_2 _+ 4 m [P_4,4,4_,(2O1)][TFSI] under different current‐density/areal‐capacity conditions. The electrolyte remains stable for >6400 h at 0.5 mA cm^−2^/0.5 mAh cm^−2^, >800 h at 1 mA cm^−2^/1 mAh cm^−2^, and >280 h at 2.5 mA cm^−2^/2.5 mAh cm^−2^. Data redrawn from Ref. [185].

These results show that ether‐functionalized phosphonium cations regulate water screening, Zn/electrolyte interfacial structure, Zn deposition uniformity, and long‐term Zn‐metal reversibility [185]. The contrast between [P_4,4,4,1_]^+^ and [P_4,4,4_,(2O1)]^+^ further indicates that ether‐functionalized or otherwise polar‐tailored phosphonium cations are more relevant for rechargeable Zn plating/stripping than purely hydrophobic long‐chain phosphonium cations [185, 189]. Phosphonium‐derived deep‐eutectic‐type electrolytes have also been investigated as low‐volatility platforms for non‐aqueous Zn–air batteries [23]. A representative methyltriphenylphosphonium bromide/ethylene glycol DES showed composition‐dependent density, viscosity, surface tension, conductivity, Walden behavior, cell voltage, peak power density, and limiting current, demonstrating that the salt‐to‐hydrogen‐bond‐donor ratio controls Zn–air electrolyte performance [23].

However, this evidence primarily supports the feasibility of phosphonium DES in Zn–air configurations and should not be interpreted as direct proof of durable Zn‐metal reversibility, long‐term Zn plating/stripping, or dendrite suppression in rechargeable Zn‐metal cells [23, 185]. Water content must be carefully controlled in phosphonium‐rich Zn electrolytes because it enhances ion transport while also altering hydrogen bonding, Zn/electrolyte interfacial reactions, corrosion behavior, hydrogen evolution, and Zn deposition chemistry [23, 35, 185]. Corrosion and current‐collector compatibility also require explicit evaluation because phosphonium IL corrosion behavior, charge transfer, pitting tendency, and adsorption mechanism can depend strongly on anion identity and metal surface chemistry [146].

Overall, phosphonium‐based electrolytes offer two complementary routes for Zn systems: functionalized phosphonium salts that regulate Zn‐metal interphases in aqueous rechargeable batteries, and phosphonium DESs that provide tunable low‐volatility media for Zn–air configurations [23, 185]. The main remaining challenges are viscosity control, Zn^2+^ speciation, standardized validation of Zn plating/stripping, hydrogen‐evolution suppression, current‐collector corrosion, and long‐term cathode compatibility under practical conditions [23, 35, 146, 185, 189]. Representative phosphonium‐based Zn electrolyte formulations are summarized in Table 2.

### 4.4 Magnesium Systems

Rechargeable magnesium batteries are attractive for large‐scale energy storage because Mg metal is earth‐abundant, low‐cost, comparatively safe to handle, and offers a high theoretical volumetric capacity of ≈3830 mAh cm^−3^ [8, 9, 17]. However, their practical development remains strongly constrained by electrolyte chemistry because Mg^2+^ exhibits high charge density, strong solvation, slow desolvation kinetics, complex solution speciation, and a tendency to form passivating interphases that hinder reversible Mg plating/stripping [8, 9]. IL‐based Mg electrolytes remain considerably less mature than their Li‐ and Na‐based counterparts, largely because these coupled transport and interfacial limitations restrict effective Mg^2+^ mobility and reversible electrochemistry [8, 9, 201]. Most IL‐based Mg studies have used Mg(TFSI)_2_ or related Mg salts, but Mg(TFSI)_2_‐containing IL electrolytes can suffer from reductive instability when [TFSI]^−^ coordinates to Mg^2+^, leading to decomposition‐derived interphases that suppress Mg‐ion transport at the Mg metal surface [8, 9].

Reported strategies to improve Mg reversibility include introducing ether or oligomeric ether donors, using ether‐functionalized IL cations, modifying the [TFSI]^−^/[FSI]^−^ ratio, applying cosolvents to reduce viscosity, and designing weakly coordinating anions or alternative Mg salts that support Mg^2+^ transport without forming blocking surface films [8, 9]. Phosphonium ILs offer a promising yet underdeveloped platform for Mg electrolytes [8, 26, 27, 30, 112]. In Mg contexts, long‐chain phosphonium salts such as [P_6,6,6,14_][Cl] are mainly useful as low‐volatility, hydrophobic, interfacially active media, but Mg^2+^ transport, Mg coordination, corrosion tendency, and surface‐film chemistry still depend strongly on the anion and formulation [8, 9, 17, 23, 202]. However, direct evidence for Mg electrolytes containing phosphonium ILs remains limited [17].

Among the currently available reports, the best‐characterized example is a primary Mg–air system based on [P_6,6,6,14_][Cl], rather than a fully rechargeable Mg plating/stripping electrolyte [17]. In [P_6,6,6,14_][Cl], Mg discharge generated a hydrated organophosphonium–magnesium hydroxyl chloride interfacial film, and the electrolyte supported oxygen‐reduction currents above 0.8 mA cm^−2^ [17, 202]. This indicates that phosphonium IL chemistry contributes to Mg/electrolyte interfacial stabilization and to cathodic oxygen chemistry under Mg–air conditions [17, 202]. However, chloride‐containing phosphonium electrolytes should not be generalized to fully rechargeable Mg‐battery electrolytes, as chloride can influence corrosion, interfacial chemistry, and long‐term reversibility [8, 9, 17]. Additional evidence that ionic liquids can chemically modify Mg‐containing surfaces comes from studies of phosphonate IL coatings on the AZ31B magnesium alloy, a commercial Mg–Al–Zn alloy [203].

In these studies, chronoamperometric treatment in 1‐ethyl‐3‐methylimidazolium ethylphosphonate ([EMIM][EtPO_3_H]) produced a 374 ± 36 nm coating containing magnesium oxide, magnesium hydroxide, magnesium carbonate, and magnesium/aluminum phosphates [203]. This evidence supports the broader concept of IL‐driven Mg surface modification, but it does not demonstrate reversible Mg plating/stripping or full battery operation [8, 203]. Therefore, phosphonium ILs are best viewed at present as tunable electrolyte components, cosolvents, additives, or interphase‐forming media for Mg‐based systems rather than as established stand‐alone rechargeable Mg‐battery electrolytes [8, 9, 14, 17, 26, 27, 30, 64, 112, 146]. Future progress will require phosphonium formulations that combine Mg^2+^ transport, controlled Mg coordination, non‐passivating interphase formation, oxidative stability at the cathode, and low corrosion toward current collectors and cell components [8, 9, 14, 17, 64, 146].

### 4.5 Aluminum Systems

Al‐based electrochemical ESSs are attractive because Al is earth‐abundant, low‐cost, relatively safe to handle, and offers a very high theoretical volumetric capacity of ≈8040 mAh cm^−3^ through the three‐electron Al^3+^/Al redox reaction [3, 8]. However, rechargeable Al batteries are strongly limited by electrolyte chemistry because reversible Al plating/stripping requires electrolytes that generate electrochemically active Al‐containing species, overcome the native Al_2_O_3_ passivation layer, minimize corrosion, and remain compatible with cathodes, current collectors, separators, and cell hardware [3, 8]. Most room‐temperature Al battery studies rely on chloroaluminate ILs such as 1‐ethyl‐3‐methylimidazolium chloride/aluminum chloride (EMImCl–AlCl_3_), or related AlCl_3_‐containing formulations because they generate [AlCl_4_]^−^ and [Al_2_Cl_7_]^−^ species that support Al deposition and graphite‐cathode anion intercalation [3, 8].

In these electrolytes, [Al_2_Cl_7_]^−^ is generally considered the key electroactive species for Al plating/stripping, while [AlCl_4_]^−^ can participate in graphite intercalation at high cathode potentials [3, 8]. This chemistry explains why chloroaluminate ILs remain difficult to replace: they can remove or bypass Al oxide passivation and enable reversible Al electrochemistry more effectively than many nonchloride molecular or ionic electrolytes [3, 8]. Nevertheless, the same chloride‐rich chemistry introduces major practical limitations, including moisture sensitivity, corrosion, side reactions, gas evolution, and restricted compatibility with current collectors, separators, binders, cell housings, and pouch‐cell hardware [3, 8]. Phosphonium ILs are therefore more realistically considered as tunable electrolyte components, co‐solvents, additives, corrosion‐modifying media, or interfacial modifiers for Al‐related electrolytes rather than direct one‐component replacements for chloroaluminate systems [14, 21, 30, 35, 69, 108].

Long‐chain phosphonium cations such as [P_6,6,6,14_]^+^ enhance hydrophobicity and reduce volatility, while the anion governs transport, electrochemical stability, corrosion tendency, interfacial film formation, and impurity sensitivity [14, 21, 30, 108]. This limitation is particularly important when interpreting halogen‐free or weakly coordinating phosphonium salts, such as phosphonium–borate systems, which should be treated as supporting‐electrolyte components unless Al speciation and reversible Al plating/stripping are directly demonstrated [3, 8, 69]. Thermally stable phosphonium–borate salts, including [P_6,6,6,14_][BOB], demonstrate the robustness, hydrophobicity, and tunability of halogen‐free phosphonium chemistry, but they should not be presented as stand‐alone Al‐battery electrolytes without direct electrochemical proof of Al deposition/stripping [69].

The high‐purity [P_6,6,6,14_][BOB] study further shows that residual water, impurities, and anion‐derived complexes can strongly affect viscosity, conductivity, thermal stability, and apparent ESW, which is especially relevant for Al systems where passivation and corrosion are impurity‐sensitive [69]. Surface and corrosion studies provide useful but indirect evidence for the possible role of phosphonium ILs in Al‐containing environments [81, 108, 204]. Studies on Al‐containing surfaces show that phosphonium cations and anions participate in surface‐film formation, tribochemical reactions, corrosion modification, or passivation processes, supporting their potential role in controlling Al/electrolyte interfacial chemistry [81, 108, 204].

However, such surface‐film or corrosion‐modification evidence must be distinguished from direct electrochemical proof of reversible Al plating/stripping in a battery electrolyte [3, 8]. Recent Al electrolyte development also shows that IL analogues and liquid coordination complexes, especially urea–AlCl_3_ and related amide–AlCl_3_ systems, provide lower‐cost routes to Al battery electrolytes while retaining Al‐chloride speciation [3]. Urea–AlCl_3_‐based electrolytes have enabled Al–graphite cycling and can show improved initial discharge capacity and Coulombic efficiency compared with some imidazolium chloroaluminate systems, although limited ionic dissociation and modest conductivity remain important barriers [3]. These systems emphasize that donor‐ligand design, AlCl_3_ coordination, and electrolyte speciation may be more important for Al batteries than simply selecting a chemically stable organic cation [3, 8]. Overall, phosphonium‐based Al electrolytes must balance electrochemical activity with chemical stability [3, 8].

Chloride‐rich or chloroaluminate‐type formulations can promote Al deposition by generating electroactive Al‐chloride complexes, but they also increase the risk of corrosion and impose strict compatibility requirements on cathodes, current collectors, separators, binders, and cell housings [3, 8, 69]. In contrast, weakly coordinating, fluorine‐free, borate‐based, or hydrophobic phosphonium salts may improve safety, thermal stability, water tolerance, and sustainability, but they will not enable reversible Al plating/stripping unless the electrolyte formulation also produces suitable Al coordination and charge‐transfer pathways [3, 8, 14, 21, 69]. Therefore, the most promising role of phosphonium ILs in Al systems is as tunable components to reduce volatility, improve hydrophobicity, modify corrosion/passivation behavior, and stabilize Al/electrolyte or cathode/electrolyte interfaces when combined with suitable Al‐coordination chemistry [3, 8, 21, 35, 69, 108, 204].

### 4.6 Cross‐System Comparison and Battery‐Specific Design Rules

Across metal‐battery chemistries, phosphonium‐based electrolytes should be selected based on the dominant cell‐level limitation rather than on generic ionic‐liquid properties alone [4, 8, 23, 69, 84]. Their function depends on the target metal, active ion, or metal‐complex speciation, the required interphase chemistry, the degradation pathway, and practical transport constraints [3, 8, 9, 10, 23]. For Li and Na systems, the strongest role of phosphonium electrolytes is interphase regulation combined with safer high‐salt, hybrid, or additive‐based electrolyte design [10, 71, 76, 84, 99]. Compact or ether‐functionalized phosphonium cations combined with [FSI]^−^/[TFSI]^−^‐rich formulations, concentrated salt structures, hybrid carbonate/IL electrolytes, or polymerizable additives are generally more promising than highly viscous neat long‐chain phosphonium ILs [15, 71, 76, 84, 99, 194].

In these systems, the key performance criteria are Li^+^ or Na^+^ transport, stable SEI/CEI formation, high Coulombic efficiency, dendrite suppression, cathode compatibility, and practical full‐cell validation [10, 71, 76, 84, 99, 194]. For Zn systems, phosphonium electrolytes follow two main routes: functionalized phosphonium salts that regulate Zn‐metal interphases in aqueous rechargeable batteries, and phosphonium‐derived DES or hybrid electrolytes that tune water activity, hydrogen bonding, and Zn–air electrolyte behavior [23, 185]. However, practical Zn‐battery relevance requires demonstration of Zn plating/stripping efficiency, suppression of hydrogen evolution, corrosion control, current‐collector compatibility, cathode stability, and long‐term cycling under standardized conditions [23, 146, 151, 185, 189].

For Mg and Al systems, the central requirement is not only oxidative stability or low volatility, but the formation of electrochemically active, non‐blocking interfacial environments [3, 8, 9, 17]. Mg electrolytes must support Mg^2+^ coordination, desolvation, and transport without forming Mg^2+^‐impermeable passivation layers [3, 8, 9, 17]. Al electrolytes must generate active Al‐containing species while suppressing oxide passivation, corrosion, and incompatibility with cell hardware [3, 8, 9]. Therefore, phosphonium ILs are best viewed today as tunable electrolyte components, co‐solvents, corrosion‐modifying media, or interfacial additives for Mg and Al systems rather than as established one‐component rechargeable metal‐battery electrolytes [3, 8, 9, 17, 69, 203, 204].

## 5 Phosphonium ILs in Supercapacitors and Quasi‐Solid Electrolytes

### 5.1 Supercapacitors

Phosphonium ILs and phosphonium salts are relevant for electrochemical capacitors because their broad apparent electrochemical stability windows, low volatility, and thermal robustness enable higher operating voltages than conventional aqueous or molecular‐solvent electrolytes [30, 94, 100]. In early activated‐carbon supercapacitors, [TFSI]¯ and [DCA]¯ salts were evaluated as capacitor electrolytes, with 5–25 wt.% AN used to reduce viscosity and improve ion transport [94]. The [P_6,6,6,14_][TFSI]/25 wt.% AN system delivered ≈100 F g^−1^ at 3.4 V with an energy density of ≈40 Wh kg^−1^ and good cycling stability [94]. These results show that phosphonium electrolytes can support high‐voltage carbon‐based capacitors, but also that reducing viscosity and improving pore accessibility are critical for practical rate performance [94].

In electric double‐layer capacitors, the ion size, ion shape, viscosity, conductivity, and packing behavior of the phosphonium electrolyte strongly influence pore accessibility, interfacial capacitance, internal resistance, and rate capability [30, 94, 100]. Therefore, supercapacitor electrolytes require a different optimization logic from metal‐battery electrolytes: fast ion adsorption/desorption, low viscosity, high conductivity, efficient pore filling, and compatibility with high‐surface‐area carbon electrodes are more important than metal‐ion solvation or reversible metal plating/stripping [94, 100]. This means that compact or asymmetric phosphonium cations, ether‐functionalized phosphonium structures, and solvent‐assisted low‐viscosity formulations are generally more attractive for high‐power capacitive storage than neat long‐chain phosphonium ILs [30, 94, 100, 136].

Future work should therefore match phosphonium‐ion size, viscosity, conductivity, solvent content, and temperature range to the carbon pore architecture, and should standardize voltage‐window testing on realistic porous electrodes while reporting capacitance retention, leakage current, floating stability, and rate capability [94, 100, 190]. Recent phosphonium‐salt electrolytes further demonstrate that cation engineering enables high‐voltage and low‐temperature capacitor operation [100]. For example, 5‐phosphinaspiro[4.4]nonane tetrafluoroborate (PSNBF_4_), dissolved in AN or PN, showed wide ESW, high ionic conductivity, and stable operation in activated‐carbon supercapacitors [100]. PSNBF_4_/AN and PSNBF_4_/PN devices retained high capacitance during floating tests up to ≈3.1–3.2 V, and low‐temperature testing at −40°C showed stable rectangular cyclic‐voltammetry profiles and high capacitance retention, especially for PSNBF_4_/PN [100].

This example indicates that small, rigid, nonclassical phosphonium cations can be useful in low‐temperature, high‐voltage capacitors when combined with suitable molecular solvents [100]. Phosphonium electrolytes may also be useful in pseudocapacitive or conducting‐polymer systems [190]. In PEDOT‐based films, tributylmethylphosphonium tosylate/water mixtures strongly swelled PEDOT: PTS: PEG, expanded the polymer interlayer distance, and increased capacitance by ≈350% through additional double‐layer capacitance [190]. This result suggests that phosphonium ILs can actively modify conducting‐polymer/electrolyte interfaces rather than acting only as passive charge carriers [190].

### 5.2 Ionogels and Polymer Gels

Beyond neat liquid electrolytes, phosphonium‐based ILs have been incorporated into polymer gels and ionogels to improve mechanical stability, leakage resistance, electrode contact, processability, and safety [4, 21, 24]. This strategy is particularly useful because neat phosphonium ILs, especially long‐chain [P_6,6,6,14_]^+^ salts, provide low volatility and thermal robustness but often suffer from high viscosity and limited ion transport [4, 21, 24]. In polymer–IL gel electrolytes, the phosphonium IL contributes nonvolatility, thermal stability, and ionic conduction, while the polymer matrix improves dimensional stability and suppresses electrolyte leakage [24]. For example, poly(vinylidene fluoride‐*co*‐hexafluoropropylene) [P(VdF‐*co*‐HFP)]‐based solid and gel polymer electrolytes containing [P_6,6,6,14_][TFSI] showed high thermal stability and enhanced ionic conductivity after plasticization with EC/PC, demonstrating how polymer–IL–plasticizer combinations can overcome the poor conductivity of dry solid polymer electrolytes [24].

### 5.3 Polymeric ILs

Polymeric phosphonium ILs provide a quasi‐solid electrolyte architecture in which phosphonium ionic groups are covalently integrated into a polymer backbone or side chain, rather than physically trapped within a neutral polymer gel [205, 206]. This distinction is important because polymeric ILs improve dimensional stability, suppress leakage, and enhance mechanical integrity while retaining tunable phosphonium cation–anion chemistry [205, 206]. Earlier oxanorbornene‐based phosphonium polymeric ILs showed that substituent structure, lithium bis(trifluoromethanesulfonyl)imide (LiTFSI) doping, and poly(ethylene glycol) incorporation strongly affect ionic conductivity, confirming that polymer architecture can tune ion transport in phosphonium‐containing solid electrolytes [65].

Phosphonium poly‐IL solid polymer electrolytes based on poly(diallyldimethylphosphonium) (PDDP) backbones further showed that [FSI]^−^/[TFSI]^−^ chemistry and high LiFSI loading enable polymer‐in‐salt conduction, with conductivities up to 1.5 × 10^−3^ S cm^−^^1^ at 80°C and Li^+^ transference numbers up to 0.7 [22, 65, 206]. These systems should be distinguished from IL–polymer blends, where a molecular phosphonium IL is physically incorporated into a neutral polymer matrix rather than covalently integrated into a polymeric ionic‐liquid structure [205, 206]. Therefore, polymeric phosphonium ILs are most relevant for solid‐state, gel‐like, or membrane‐based electrolyte architectures in which mechanical stability, leakage resistance, and interfacial compatibility take precedence over maximum room‐temperature ionic conductivity [22, 65, 205, 206].

### 5.4 DES‐Type and Hybrid Electrolyte Systems

Phosphonium‐based deep‐eutectic‐type and hybrid electrolyte systems provide an alternative route to tune electrolyte properties when neat phosphonium ILs are too viscous for high‐rate operation [23, 69, 207]. In these systems, phosphonium salts are combined with hydrogen‐bond donors, molecular solvents, polymer matrices, or other ionic components to adjust melting behavior, viscosity, conductivity, surface tension, electrode wetting, and interfacial compatibility [23, 69, 207]. This strategy is particularly useful because it can preserve some advantages of phosphonium chemistry, such as low volatility, thermal robustness, and tunable cation–anion interactions, while improving processability and ion transport [4, 23, 35, 69].

Representative phosphonium DES‐type systems include methyltriphenylphosphonium bromide/ethylene glycol mixtures for non‐aqueous Zn–air batteries, where the salt‐to‐hydrogen‐bond‐donor ratio controlled the density, viscosity, surface tension, conductivity, Walden behavior, cell voltage, power density, and limiting current [23]. This example shows that phosphonium DES chemistry provides a tunable electrolyte platform, but the evidence mainly supports Zn–air operation rather than durable rechargeable Zn plating/stripping [23, 185]. More broadly, phosphonium/ammonium salt mixtures with glycerol or related hydrogen‐bond donors demonstrate that DES‐type electrolyte properties are governed by coupled effects of donor identity, water content, salt concentration, and operating temperature [23, 207].

Hybrid phosphonium electrolytes also include solvent‐assisted, gel/polymer, and quasi‐solid formulations that use molecular solvents, plasticizers, or polymer networks to reduce viscosity, improve electrode contact, suppress leakage, and enhance mechanical stability [4, 24, 69, 98]. Compared with neat long‐chain phosphonium ILs, these formulations can improve conductivity and processability, but their electrochemical window, thermal stability, metal compatibility, and long‐term cycling behavior remain strongly composition‐dependent [4, 24, 69, 98]. Therefore, DES‐type and hybrid phosphonium electrolytes should be treated as composition‐sensitive formulations rather than universally superior alternatives to neat ILs; their value depends on controlling hydrogen‐bond‐donor content, water level, solvent fraction, polymer fraction, viscosity, conductivity, and interfacial chemistry for the target electrode system [4, 23, 24, 69, 98].

### 5.5 Practical Design Rules, Sustainability, and Standardized Benchmarking

Despite their promising safety profile and tunable chemistry, phosphonium‐based electrolytes face several practical barriers, including high viscosity in long‐chain systems, dependence on fluorinated anions, cost, synthesis complexity, impurity sensitivity, and inconsistent benchmarking protocols [18, 30, 69]. Fluorinated anions such as [FSI]¯ and [TFSI]¯ often offer favorable conductivity, salt compatibility, apparent electrochemical stability, and the formation of an inorganic‐rich interphase, but they raise concerns about fluorine content, environmental persistence, cost, and end‐of‐life management [18, 63]. Fluorine‐reduced or fluorine‐free alternatives, including [DCA]¯, [BOB]¯, carboxylates, oxalates, and borate‐based anions, can improve sustainability but may also change viscosity, conductivity, voltage stability, corrosion behavior, and interfacial chemistry [63, 69, 106].

Therefore, sustainability cannot be evaluated only by removing fluorine; it must also consider electrolyte lifetime, synthesis route, purity requirements, recyclability, toxicity, safety, and full‐cell performance [18, 31]. Table 3 summarizes the main design targets, preferred phosphonium‐electrolyte strategies, expected benefits, and remaining challenges identified throughout this Review [18, 69]. It is intended as a design guide rather than a universal ranking because phosphonium‐electrolyte performance is strongly system‐ and protocol‐dependent [18, 27, 30, 69, 191]. A major remaining limitation is the lack of standardized reporting across phosphonium‐electrolyte studies [27, 30].

**TABLE 3:** Practical design rules and remaining challenges for phosphonium‐based electrolytes. The table summarizes key design targets, preferred phosphonium‐electrolyte strategies, expected benefits, and unresolved limitations across battery, supercapacitor, gel, polymeric‐IL, and DES‐type electrolyte formats.

Design target	Preferred phosphonium strategy	Main benefit	Remaining challenge
High‐rate ion transport	Short‐chain, asymmetric, branched, ether‐functionalized cations	Improved ion mobility	Possible loss of oxidative/interfacial stability
Thermal/volatility control	Long‐chain hydrophobic cations; stable anions	Low volatility, high thermal stability	High viscosity, poor rate capability
Fluorine‐reduced sustainability	[DCA]^−^, [BOB]^−^, carboxylates, borates	Reduced fluorinated‐anion concern	Possible loss of conductivity or voltage stability
Li/Na metal stability	[FSI]^−^/[TFSI]^−^ or concentrated phosphonium electrolytes	LiF/NaF‐rich SEI, improved plating	Cost, viscosity, salt concentration sensitivity
Zn systems	Ether‐functionalized phosphonium salts; DES/hybrid phosphonium formulations	Lower volatility, tunable water activity	Limited standardized practical Zn validation
Mg/Al systems	Mg/Al‐speciation‐controlled systems	Metal‐ion/metal‐complex speciation control	Passivation/corrosion problems
Supercapacitors	Compact cations or solvent‐assisted formulations	High voltage, low‐temperature operation	Ion size and pore accessibility
Gel/DES systems	Polymer/phosphonium IL or DES hybrids	Leakage resistance, safety	Composition and water/HBD sensitivity

Abbreviations: HBD, hydrogen‐bond donor.

Apparent ESWs are often measured using different working electrodes, reference scales, scan rates, temperatures, cutoff‐current criteria, and water/halide contents, while conductivity and viscosity are frequently reported at different temperatures or salt concentrations [27, 30, 208]. These inconsistencies make direct comparison difficult and can lead to misleading rankings, especially when apparent ESW values or thermal‐decomposition temperatures are treated as absolute operating limits [69, 208]. Future reports should provide, at a minimum, the cation/anion structure, electrolyte purity, water and halide content, salt concentration, temperature, viscosity, conductivity, electrochemical‐window protocol, electrode materials, cell configuration, electrolyte amount, cycling conditions, and post‐cycling interfacial analysis [18, 208].

### 5.6 Electrolyte Behavior Under Extreme Operating Conditions

Phosphonium‐electrolyte design becomes particularly demanding under high‐rate, low‐temperature, and high‐voltage operation because the dominant limitations shift with operating conditions. At high current density, concentration gradients, sluggish desolvation, and ion transport through the interphase can produce large polarization even when room‐temperature bulk conductivity is acceptable [70, 159]. Compact, asymmetric, branched, or ether‐functionalized phosphonium cations are therefore generally more favorable for high‐rate operation because they reduce viscosity and improve ion mobility [26, 123]. Concentrated [FSI]^−^‐based formulations can additionally stabilize metal deposition through inorganic‐rich interphases [70, 135, 159].

At low temperature, the sharp increase in viscosity and reduction in ionic mobility can become the primary limitation [30]. This favors compact, asymmetric, or appropriately functionalized low‐melting phosphonium cations, weakly coordinating anions, and solvent‐assisted or hybrid formulations that suppress crystallization and maintain sufficient conductivity [26, 30, 123]. Recent phosphonium‐salt supercapacitor electrolytes demonstrate that appropriate cation and solvent selection can preserve capacitive response even at −40°C, showing that phosphonium chemistry can be adapted to sub‐zero operation when transport rather than thermal stability is prioritized [100, 135]. More direct battery‐level evidence has recently been reported for sub‐zero phosphonium‐electrolyte design.

A phosphonium‐based ionic‐liquid additive was shown to form a eutectic environment with a carbonate solvent, reducing freezing tendency and viscosity while weakening Li^+^–solvent coordination. The associated improvement in Li^+^ desolvation and formation of a thin LiF‐rich SEI enabled high‐loading NCM811‖graphite pouch cells to retain approximately 88% of their capacity after 100 cycles at −20°C [209]. This result shows that low‐temperature phosphonium‐electrolyte design should address both bulk phase behavior and interfacial desolvation rather than viscosity alone. High‐voltage operation imposes a different constraint because oxidative electrolyte decomposition and CEI formation become dominant [155].

In this regime, increasing the apparent electrochemical stability window alone is insufficient; the electrolyte must also form a stable cathode/electrolyte interface and resist continuous oxidation at practical electrode potentials [161]. Phosphonium electrolytes containing oxidation‐resistant anions or carefully designed hybrid formulations have enabled operation with high‐voltage positive electrodes, but this stability must be balanced against viscosity, salt dissociation, and interfacial resistance [75]. These trade‐offs indicate that no single phosphonium cation–anion pair is optimal across all operating extremes, and molecular design should instead target the dominant kinetic or interfacial failure mode of the intended application [70, 75].

### 5.7 Summary of Design Rules

The design rules developed above are summarized in Figure 9 as a practical roadmap from molecular electrolyte design to application‐specific validation [3, 4, 14, 18, 26, 27, 30, 63, 69, 84, 98]. Beyond molecular design, practical phosphonium‐electrolyte performance depends on electrolyte format, purity, and benchmarking conditions. Neat phosphonium ILs are not always the most practical electrolytes; hybrid carbonate–IL systems, super concentrated salt–IL electrolytes, polymer gels, polymeric ILs, and DES‐type formulations may provide better performance for specific cell chemistries [18, 27, 71, 84]. Therefore, phosphonium electrolytes should be evaluated based on the combined requirements of ion transport, interphase control, corrosion suppression, gas/thermal safety, and long‐term compatibility with the target electrode chemistry, rather than on apparent ESW or bulk conductivity alone [63, 71, 84, 188].

**FIGURE 9:**
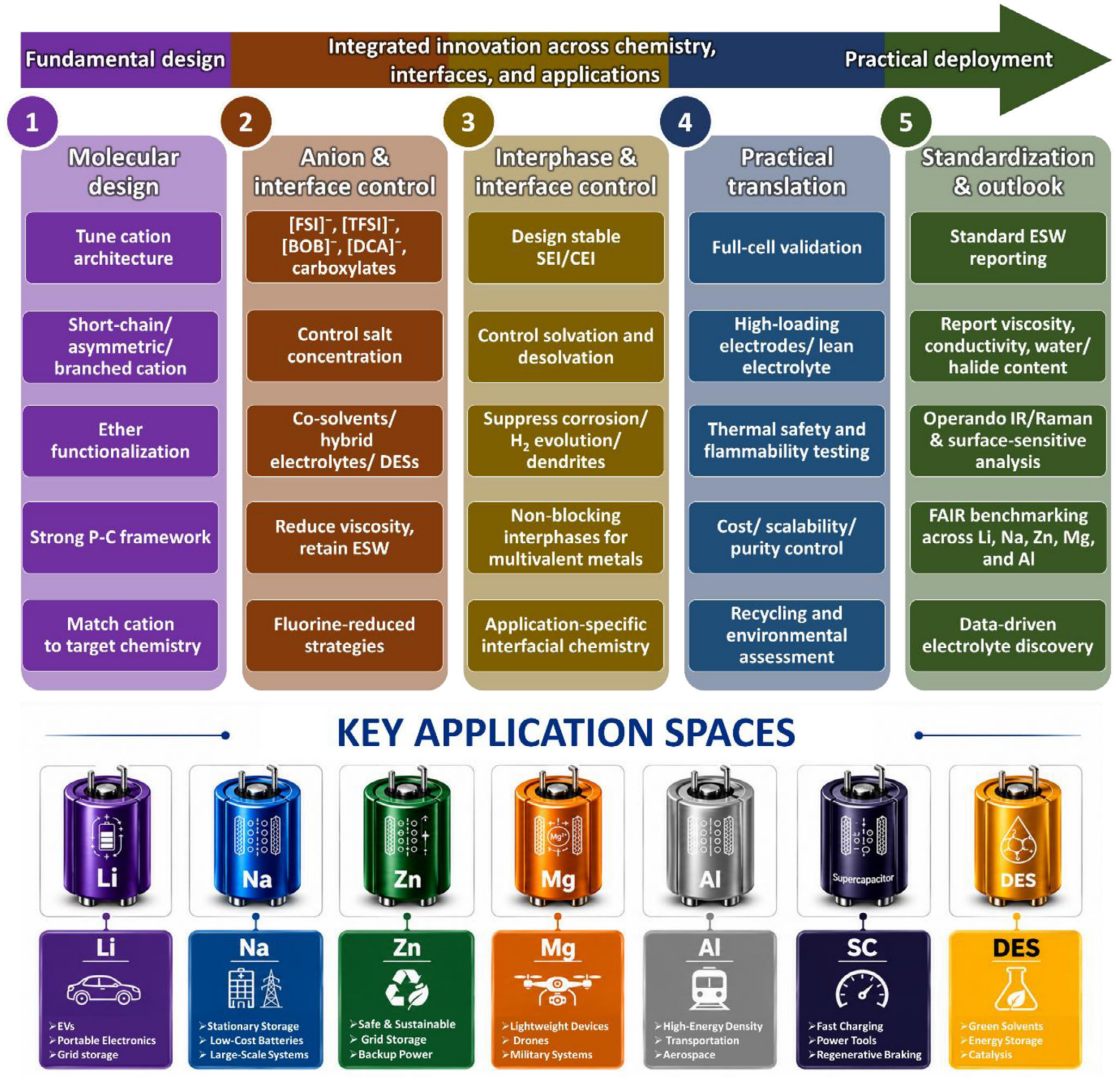
Roadmap for phosphonium‐electrolyte design, linking molecular cation engineering, anion/electrolyte control, interphase regulation, practical full‐cell validation, and standardized benchmarking. Major targets include low viscosity, fast ion transport, broad apparent ESW, stable SEI/CEI formation, improved safety, and more sustainable electrolyte design across Li‐, Na‐, Zn‐, Mg‐, and Al‐based systems, as well as supercapacitor and hybrid‐electrolyte systems. Operando/in situ IR methods, including ATR‐FTIR, SEIRAS, and PM‐IRRAS, are included as complementary probes of adsorption, molecular orientation, solvation, ion pairing, and the onset and evolution of interphase formation [180, 181, 182]. The figure was created by the authors based on design principles and literature trends discussed in this Review [3, 4, 14, 18, 26, 27, 30, 63, 69, 84, 98]. Abbreviations: EVs, electric vehicles; FAIR, Findable, Accessible, Interoperable, and Reusable; SC, supercapacitor; DES, deep eutectic solvent. IR, infrared; ATR‐FTIR, attenuated total reflection Fourier‐transform infrared spectroscopy; SEIRAS, surface‐enhanced infrared absorption spectroscopy; PM‐IRRAS, polarization‐modulation infrared reflection–absorption spectroscopy.

The reviewed literature shows that phosphonium‐based electrolytes should be designed by balancing four coupled parameters: cation architecture, anion coordination, electrolyte formulation, and interfacial reactivity. Compact, branched, asymmetric, or ether‐functionalized phosphonium cations are generally preferred when low viscosity, high ionic conductivity, and fast ion transport are required, whereas long‐chain hydrophobic cations such as [P_6,6,6,14_]^+^ are more useful when low volatility, hydrophobicity, thermal robustness, or gel/DES formulation stability is prioritized. Anion selection must be matched to the target chemistry rather than optimized only by the apparent ESW. [FSI]¯ and [TFSI]¯ often provide favorable transport, salt compatibility, and inorganic‐rich interphase formation, but fluorine‐reduced or fluorine‐free alternatives such as [DCA]¯, [BOB]¯, carboxylates, oxalates, and borate‐based species are increasingly important for sustainability. Sustainability must be assessed together with lifetime, synthesis route, recyclability, toxicity, safety, and full‐cell performance.

## 6 Conclusion and Outlook

Phosphonium ILs represent a versatile yet underexploited electrolyte platform for safer, more durable electrochemical energy storage systems. Their value does not lie in universally replacing conventional electrolytes, but in enabling application‐specific control over ion transport, solvation, interphase formation, corrosion, volatility, and thermal safety. The design rules emerging from this Review show that phosphonium electrolytes should be optimized through the coupled selection of cation architecture, anion chemistry, electrolyte format, purity, and electrode chemistry. Compact, asymmetric, branched, or ether‐functionalized cations are most attractive when fast ion transport is required, whereas long‐chain hydrophobic cations are better suited for low‐volatility, thermally robust, gel, or DES‐type formulations.

At the same time, [FSI]^−^/[TFSI]^−^‐based systems must increasingly be balanced against fluorine‐reduced or fluorine‐free alternatives, standardized benchmarking, full‐cell validation, and surface‐sensitive or operando interphase characterization. Future progress will therefore depend on moving beyond apparent ESW or bulk conductivity alone and toward integrated evaluation of transport, interphase chemistry, safety, sustainability, and practical cell performance. With continued advances in molecular design, standardized benchmarking, and mechanistic interphase characterization, phosphonium ILs are well‐positioned to become a promising and highly tunable electrolyte platform for next‐generation electrochemical energy‐storage systems.

## Author Contributions

**Amir Haghipour**: conceptualization, data curation, formal analysis, visualization, project administration, writing the original draft, and writing the review and editing. **Ishanka Thennakoon**: writing the review and editing, and data curation. **Aishwaryaba Jadeja**: writing the review and editing. **Daniel Rauber**: writing the review and editing. **Markus Gallei**: writing the review and editing. **Rolf Hempelmann**: writing the review and editing. **Sangwon Kim**: writing the review and editing. **Jungtae Kim**: funding acquisition, supervision, and writing the review and editing.

## Conflicts of Interest

The authors declare no conflicts of interest.

## Supporting information




**Supporting File**: advs78370‐sup‐0001‐SuppMat.docx.

## Data Availability

All data discussed in this review are derived from published literature and are cited in the article and Supplementary Information.
