# Supplementary material for: Phosphonium Ionic Liquids for Electrochemical Energy Storage: Structure–Property–Interphase Relationships and Design Rule
Source: Adv Sci (Weinh). 2026 Oct 22:e78370. Online ahead of print. doi: 10.1002/advs.78370 (PMC13691243; doi:10.1002/advs.78370)
Supplement: Supplementary file 1 [file ADVS-9999-e78370-s001.docx]

Supplemental information (SI) for

## Phosphonium Ionic Liquids for Electrochemical Energy Storage: Structure–Property–Interphase Relationships and Design Rules

## Amir Haghipour,^1^ Ishanka Thennakoon,^1^ Aishwaryaba Jadeja,^1^ Daniel Rauber,^2,3^ Markus Gallei,^2,3^ Rolf Hempelmann,^4^ Sangwon Kim^1,4^, Jungtae Kim^1*^

1- Korea Institute of Science and Technology (KIST) Europe, Saarbrücken, Germany
2- Saarland University, Polymer Chemistry, Campus C4 2, 66123 Saarbrücken, Germany.
3- Saarene, Saarland Center for Energy Materials and Sustainability, Campus C4 2, 66123 Saarbrücken, Germany
4- Transfercenter Sustainable Electrochemistry, Saarland University, 66123 Saarbrücken, Germany

****Corresponding author:*** *Jungtae Kim;* ***Email:*** *tais@kist-europe.de*

**Table S1.** Literature-derived electrochemical window dataset for representative ionic liquids (ILs) grouped by cation family. The table summarizes reported electrochemical windows (EW), cathodic and anodic stability limits, scan rates, cutoff current densities, electrode/reference configurations, and experimental conditions for the ILs including, ammonium, imidazolium, morpholinium, phosphonium, piperidinium, pyrazolium, pyridinium, pyrrolidinium, and sulfonium. Because ESW values are strongly protocol-dependent, the listed values should be interpreted as comparative literature trends rather than absolute intrinsic stability limits; differences in electrode material, reference scale, cutoff criterion, scan rate, temperature, solvent/additive content, water or impurity level, and experimental versus computational methodology can shift the apparent stability window.

| **Cation Family** | **Ionic Liquid** | **App. EW/ESW (V)** | $\boldsymbol{E}_{\boldsymbol{cat}}$ **(V)** | $\boldsymbol{E}_{\boldsymbol{an}}$  **(V)** | **ν (mV s^-1^)** | $\boldsymbol{j}_{\boldsymbol{cut}}$**(mA cm^-2^)** | **Electrode/reference and conditions** | **Ref.** |
| --- | --- | --- | --- | --- | --- | --- | --- | --- |
| **Amm.** | **tributylmethylammonium bis(trifluoromethylsulfonyl)imide** | **5.2** | **-3.5** | **1.7** | **100** | **0.50** | **Pt microelectrode.** | ^[1]^ |
| **Amm.** | **trimethylpropylammonium bis(trifluoromethylsulfonyl)imide** | **5.6** | **-3.0** | **2.6** | **50** | **0.50** | **GC electrode.** | ^[1]^ |
| **Amm.** | **trimethylmethoxymethylammonium bis(trifluoromethylsulfonyl)imide** | **5.2** | **-2.7** | **2.5** | **50** | **0.50** | **GC electrode.** | ^[1]^ |
| **Amm.** | **trimethylphenylammonium bis(trifluoromethylsulfonyl)imide** | **4.6** | **-2.3** | **2.3** | **50** | **0.50** | **GC electrode.** | ^[1]^ |
| **Amm.** | **tetraethylammonium 2,2,2-trifluoro-N-(trifluoromethylsulfonyl)acetamide** | **4.6** | **-2.4** | **2.2** | **50** | **0.50** | **GC electrode.** | ^[1]^ |
| **Amm.** | **n-hexyltriethylammonium bis(trifluoromethylsulfonyl)imide** | **5.7 - 5.9** | **NR** | **NR** | **100** | **NR** | **0.1 M IL in Acetonitrile (Au/Pt microdisks).** | ^[2]^ |
| **Amm.** | **N-ethyl-N,N-dimethyl-2-methoxyethylammonium bis(trifluoromethylsulfonyl)imide** | **5.99** | **-3.47** | **2.52** | **100** | **0.50** | **GC macro-electrode.** | ^[3]^ |
| **Amm.** | **butyltrimethylammonium bis(trifluoromethylsulfonyl)imide** | **5.9** | **NR** | **NR** | **100** | **1.91** | **Pt thin-film, <1 RH%, 15 µA cutoff.** | ^[4]^ |
| **Amm.** | **N-hexyltriethylammonium bis(trifluoromethylsulfonyl)imide** | **4.7** | **-2.6** | **2.1** | **100** | **1.00** | **Vacuum-dried, 1 mA cm^-2^ cutoff.** | ^[5]^ |
| **Amm.** | **diethanolammonium acetate** | **2.43** | **-1.84** | **0.59** | **100** | **NR** | **GC electrode (V vs. Fc0/+).** | ^[6]^ |
| **Amm.** | **diethanolammonium hydrogen sulfate** | **2.16** | **-1.05** | **1.11** | **100** | **NR** | **GC electrode (V vs. Ag wire).** | ^[6]^ |
| **Amm.** | **di-n-propylammonium formate** | **2.69** | **-2.46** | **0.23** | **100** | **NR** | **GC electrode (V vs. Fc0/+).** | ^[6]^ |
| **Amm.** | **triethylammonium acetate** | **3.42** | **-2.77** | **0.65** | **100** | **NR** | **GC electrode (V vs. Fc0/+).** | ^[6]^ |
| **Amm.** | **diethanolammonium formate** | **3.60** | **-3.39** | **0.21** | **100** | **NR** | **GC electrode (V vs. Fc0/+).** | ^[6]^ |
| **Amm.** | **triethylammonium formate** | **3.43** | **-3.04** | **0.39** | **100** | **NR** | **GC electrode (V vs. Fc0/+).** | ^[6]^ |
| **Amm.** | **diethanolammonium sulfamate** | **4.25** | **-2.41** | **1.84** | **100** | **NR** | **GC electrode (V vs. Ag wire).** | ^[6]^ |
| **Amm.** | **diethanolammonium chloride** | **4.01** | **-2.54** | **1.47** | **100** | **NR** | **GC electrode (V vs. Ag wire).** | ^[6]^ |
| **Amm.** | **tributylmethylammonium bis(trifluoromethylsulfonyl)imide** | **5.50** | **-4.01** | **1.49** | **100** | **1.00** | **GC WE; Pt CE; Ag/Ag^+^ RE; dried/N_2_-bubbled.** | ^[7]^ |
| **Amm.** | **tetraethylammonium acetate** | **4.52** | **-3.50** | **1.02** | **100** | **1.00** | **GC WE; Pt CE; Ag/Ag^+^ RE; dried/N_2_-bubbled.** | ^[7]^ |
| **Amm.** | **trimethyl-isobutyl-ammonium bis(trifluoromethylsulfonyl)imide** | **6.02** | **5.76** | **-0.26** | **2** | **0.05** | **Pt WE; Li CE/QRE; GF separator; 20 °C; vs Li^+^/Li.** | ^[8]^ |
| **Amm.** | **trimethyl-isobutyl-ammonium (fluorosulfonyl)(trifluoromethylsulfonyl)imide** | **5.78** | **5.67** | **-0.11** | **2** | **0.05** | **Pt WE; Li CE/QRE; GF separator; 20 °C; vs Li^+^/Li.** | ^[8]^ |
| **Amm.** | **trimethyl-isobutyl-ammonium bis(fluorosulfonyl)imide** | **5.73** | **5.66** | **-0.07** | **2** | **0.05** | **Pt WE; Li CE/QRE; GF separator; 20 °C; vs Li^+^/Li.** | ^[8]^ |
| **Imid.** | **1,2-dimethyl-3-propylimidazolium** | **2.2** | **0.3** | **2.5** | **20** | **0.20** | **Pt working electrode.** | ^[1]^ |
| **Imid.** | **1,2-dimethyl-3-propylimidazolium** | **2.1** | **0.2** | **2.3** | **100** | **0.10** | **Tungsten (W) electrode.** | ^[1]^ |
| **Imid.** | **1,2-dimethyl-3-propylimidazolium** | **4.7** | **-2.2** | **2.5** | **20** | **NR** | **GC working electrode.** | ^[1]^ |
| **Imid.** | **1,2-dimethyl-3-propylimidazolium** | **3.5** | **-2.4** | **1.0** | **20** | **0.10** | **Pt working electrode.** | ^[1]^ |
| **Imid.** | **1-propyl-3-methylimidazolium** | **2.7** | **NR** | **NR** | **100** | **NR** | **W working electrode.** | ^[1]^ |
| **Imid.** | **1-ethyl-3-methylimidazolium** | **2.5** | **-0.2** | **2.3** | **100** | **NR** | **W working electrode.** | ^[1]^ |
| **Imid.** | **1-ethyl-3-methylimidazolium** | **4.6** | **-2.2** | **2.4** | **20** | **NR** | **GC working electrode.** | ^[1]^ |
| **Imid.** | **1,2-dimethyl-3-ethylimidazolium bis(trifluoromethylsulfonyl)imide** | **4.9** | **-2.6** | **2.3** | **50** | **1.00** | **Pt electrode (V vs. RE).** | ^[1]^ |
| **Imid.** | **1,2-dimethyl-3-propylimidazolium bis(trifluoromethylsulfonyl)imide** | **5.4** | **1.0** | **5.4** | **20** | **1.00** | **GC electrode with Li QRE.** | ^[1]^ |
| **Imid.** | **1-ethyl-3-methylimidazolium trifluoromethanesulfonate** | **4.2** | **-2.0** | **2.2** | **50** | **NR** | **Pt electrode (10 µA cutoff).** | ^[1]^ |
| **Imid.** | **1-ethyl-3-methylimidazolium methanesulfonate** | **4.1** | **-2.0** | **2.1** | **50** | **NR** | **Pt electrode (10 µA cutoff).** | ^[1]^ |
| **Imid.** | **1-ethyl-3-methylimidazolium tetrafluoroborate** | **4.4** | **-2.0** | **2.4** | **100** | **NR** | **Pt electrode.** | ^[1]^ |
| **Imid.** | **1-ethyl-3-methylimidazolium bis(trifluoromethylsulfonyl)imide** | **4.6** | **-2.1** | **2.5** | **50** | **1.00** | **Pt electrode.** | ^[1]^ |
| **Imid.** | **1-ethyl-3-methylimidazolium bis(trifluoromethylsulfonyl)amide** | **3.8** | **-1.8** | **2.0** | **100** | **0.10** | **Pt electrode.** | ^[1]^ |
| **Imid.** | **1-ethyl-3-methylimidazolium trifluoroacetate** | **3.5** | **-2.1** | **1.4** | **50** | **1.00** | **Pt electrode.** | ^[1]^ |
| **Imid.** | **1-ethyl-3-methylimidazolium fluorohydrogenate** | **3.9** | **-1.9** | **2.1** | **10** | **0.50** | **GC electrode.** | ^[1]^ |
| **Imid.** | **1-ethyl-3-methylimidazolium 2,2,2-trifluoro-N-(trifluoromethylsulfonyl)acetamide** | **4.1** | **-2.1** | **2.0** | **50** | **0.50** | **GC electrode.** | ^[1]^ |
| **Imid.** | **1-ethyl-3-methylimidazolium hexafluorotantalate** | **4.5** | **-2.3** | **2.2** | **50** | **0.50** | **Pt electrode.** | ^[1]^ |
| **Imid.** | **1-ethyl-3-methylimidazolium bis(perfluoroethylsulfonyl)imide** | **4.1** | **-1.9** | **2.2** | **50** | **0.50** | **GC electrode.** | ^[1]^ |
| **Imid.** | **1-ethyl-3-methylimidazolium dicyanamide** | **3.1** | **-1.9** | **1.2** | **50** | **0.10** | **Pt electrode.** | ^[1]^ |
| **Imid.** | **1,3-dimethylimidazolium fluorohydrogenate** | **3.4** | **-1.7** | **1.7** | **10** | **0.50** | **GC electrode.** | ^[1]^ |
| **Imid.** | **1-propyl-3-methylimidazolium fluorohydrogenate** | **4.0** | **-1.9** | **2.1** | **10** | **0.50** | **GC electrode.** | ^[1]^ |
| **Imid.** | **1-propyl-3-methylimidazolium tetrafluoroborate** | **4.6** | **-2.2** | **2.4** | **100** | **0.50** | **GC electrode.** | ^[1]^ |
| **Imid.** | **1-butyl-3-methylimidazolium hexafluorophosphate** | **3.7** | **-2.0** | **1.7** | **100** | **0.50** | **Pt microelectrode.** | ^[1]^ |
| **Imid.** | **1-butyl-3-methylimidazolium fluorohydrogenate** | **4.1** | **-1.9** | **2.2** | **10** | **0.50** | **GC electrode.** | ^[1]^ |
| **Imid.** | **1-butyl-3-methylimidazolium tetrafluoroborate** | **4.4** | **-1.8** | **2.6** | **100** | **0.02** | **Pt microelectrode.** | ^[1]^ |
| **Imid.** | **1-pentyl-3-methylimidazolium fluorohydrogenate** | **4.2** | **-2.1** | **2.1** | **10** | **0.50** | **GC electrode.** | ^[1]^ |
| **Imid.** | **1-hexyl-3-methylimidazolium fluorohydrogenate** | **4.5** | **-2.4** | **2.1** | **10** | **0.50** | **GC electrode.** | ^[1]^ |
| **Imid.** | **1-ethyl-3-methylimidazolium bis(trifluoromethylsulfonyl)imide** | **5.4 - 5.6** | **NR** | **NR** | **100** | **NR** | **0.1 M IL in Acetonitrile (Au/Pt microdisks).** | ^[2]^ |
| **Imid.** | **1-hexyl-3-methylimidazolium trifluorotris(pentafluoroethyl)phosphate** | **5.7 - 6.0** | **NR** | **NR** | **100** | **NR** | **0.1 M IL in Acetonitrile (Au/Pt microdisks).** | ^[2]^ |
| **Imid.** | **1-ethyl-3-methylimidazolium bis(trifluoromethylsulfonyl)imide** | **4.19** | **-2.07** | **2.12** | **100** | **0.50** | **GC macro-electrode.** | ^[3]^ |
| **Imid.** | **1-ethyl-3-methylimidazolium bis(trifluoromethylsulfonyl)imide** | **4.3** | **NR** | **NR** | **100** | **1.91** | **Pt thin-film, <1 RH%, 15 µA cutoff.** | ^[4]^ |
| **Imid.** | **1-butyl-3-methylimidazolium tetrafluoroborate** | **4.4** | **NR** | **NR** | **100** | **1.91** | **Pt thin-film, <1 RH%, 15 µA cutoff.** | ^[4]^ |
| **Imid.** | **1-butyl-3-methylimidazolium hexafluorophosphate** | **4.8** | **NR** | **NR** | **100** | **1.91** | **Pt thin-film, <1 RH%, 15 µA cutoff.** | ^[4]^ |
| **Imid.** | **1-butyl-3-methylimidazolium bis(trifluoromethylsulfonyl)imide** | **4.7** | **NR** | **NR** | **100** | **1.91** | **Pt thin-film, <1 RH%, 15 µA cutoff.** | ^[4]^ |
| **Imid.** | **1-butyl-3-methylimidazolium tris(pentafluoroethyl)trifluorophosphate** | **5.2** | **NR** | **NR** | **100** | **1.91** | **Pt thin-film, <1 RH%, 15 µA cutoff.** | ^[4]^ |
| **Imid.** | **1-hexyl-3-methylimidazolium tris(perfluoroethyl)trifluorophosphate** | **4.6** | **-2.6** | **2.0** | **100** | **1.00** | **Vacuum-dried, 1 mA cm^-2^ cutoff.** | ^[5]^ |
| **Imid.** | **1-butyl-3-methylimidazolium bis(trifluoromethylsulfonyl)imide** | **4.3** | **-2.5** | **1.8** | **100** | **1.00** | **Vacuum-dried, 1 mA cm^-2^ cutoff.** | ^[5]^ |
| **Imid.** | **1-butyl-3-methylimidazolium bis-(trifluoromethylsulfonyl)imide** | **4.7** | **-2.5** | **2.2** | **100** | **1.00** | **Vacuum-dried, 1 mA cm^-2^ cutoff.** | ^[5]^ |
| **Imid.** | **1-butyl-3-methylimidazolium hexafluorophosphate** | **4.8** | **-2.7** | **2.1** | **100** | **1.00** | **Vacuum-dried, 1 mA cm^-2^ cutoff.** | ^[5]^ |
| **Imid.** | **1-ethyl-3-methylimidazolium bis(trifluoromethylsulfonyl)imide** | **4.2** | **-2.2** | **2.0** | **100** | **1.00** | **Vacuum-dried, 1 mA cm^-2^ cutoff.** | ^[5]^ |
| **Imid.** | **1-butyl-3-methylimidazolium tetrafluoroborate** | **4.6** | **-2.6** | **2.0** | **100** | **1.00** | **Vacuum-dried, 1 mA cm^-2^ cutoff.** | ^[5]^ |
| **Imid.** | **1-hexyl-3-methylimidazolium iodide** | **2.0** | **-2.0** | **0.0** | **100** | **1.00** | **Vacuum-dried, 1 mA cm^-2^ cutoff.** | ^[5]^ |
| **Imid.** | **1-butyl-3-methylimidazolium trifluoromethylsulfonate** | **4.2** | **-2.6** | **1.6** | **100** | **1.00** | **Vacuum-dried, 1 mA cm^-2^ cutoff.** | ^[5]^ |
| **Imid.** | **1-hexyl-3-methylimidazolium chloride** | **3.0** | **-2.6** | **0.4** | **100** | **1.00** | **Vacuum-dried, 1 mA cm^-2^ cutoff.** | ^[5]^ |
| **Imid.** | **1-(2-hydroxyethyl)-3-methylimidazolium bis(trifluoromethylsulfonyl)imide** | **4.27** | **-2.21** | **2.06** | **NR** | **1.00** | **Vacuum-dried, 1 mA cm^-2^ cutoff.** | ^[9]^ |
| **Imid.** | **1-(2-methoxyphenyl)-3-butylimidazolium bis(trifluoromethylsulfonyl)imide** | **3.87** | **-2.43** | **1.44** | **100** | **1.00** | **GC WE; Pt CE; Ag/Ag^+^ QRE; vs Fc/Fc^+^.** | ^[9]^ |
| **Imid.** | **1-(4-methoxyphenyl)-3-butylimidazolium bis(trifluoromethylsulfonyl)imide** | **3.28** | **-2.33** | **0.96** | **100** | **1.00** | **GC WE; Pt CE; Ag/Ag^+^ QRE; vs Fc/Fc^+^.** | ^[10]^ |
| **Imid.** | **1-(2-methylphenyl)-3-butylimidazolium bis(trifluoromethylsulfonyl)imide** | **4.11** | **-2.33** | **1.78** | **100** | **1.00** | **GC WE; Pt CE; Ag/Ag^+^ QRE; vs Fc/Fc^+^.** | ^[10]^ |
| **Imid.** | **1-(4-methylphenyl)-3-butylimidazolium bis(trifluoromethylsulfonyl)imide** | **3.39** | **-2.13** | **1.26** | **100** | **1.00** | **GC WE; Pt CE; Ag/Ag^+^ QRE; vs Fc/Fc^+^.** | ^[10]^ |
| **Imid.** | **1-(2-methoxyphenyl)-3-butylimidazolium dicyanamide** | **7.17** | **-2.42** | **4.75** | **100** | **1.00** | **GC WE; Pt CE; Ag/Ag^+^ QRE; vs Fc/Fc^+^.** | ^[10]^ |
| **Imid.** | **1-(4-methoxyphenyl)-3-butylimidazolium dicyanamide** | **5.93** | **-2.47** | **3.46** | **100** | **1.00** | **GC WE; Pt CE; Ag/Ag^+^ QRE; vs Fc/Fc^+^.** | ^[10]^ |
| **Imid.** | **1-(2-methylphenyl)-3-butylimidazolium dicyanamide** | **5.51** | **-2.53** | **2.98** | **100** | **1.00** | **GC WE; Pt CE; Ag/Ag^+^ QRE; vs Fc/Fc^+^.** | ^[10]^ |
| **Imid.** | **1-(4-methylphenyl)-3-butylimidazolium dicyanamide** | **5.41** | **-2.45** | **2.96** | **100** | **1.00** | **GC WE; Pt CE; Ag/Ag^+^ QRE; vs Fc/Fc^+^.** | ^[10]^ |
| **Imid.** | **1-ethyl-3-methylimidazolium bis(trifluoromethylsulfonyl)imide** | **4.70** | **-2.46** | **2.24** | **100** | **1.00** | **GC WE; Pt CE; Ag/Ag^+^ RE; dried/N_2_-bubbled.** | ^[7]^ |
| **Imid.** | **1-butyl-3-methylimidazolium bis(trifluoromethylsulfonyl)imide** | **5.07** | **-2.63** | **2.44** | **100** | **1.00** | **GC WE; Pt CE; Ag/Ag^+^ RE; dried/N_2_-bubbled.** | ^[7]^ |
| **Imid.** | **1-hexyl-3-methylimidazolium bis(trifluoromethylsulfonyl)imide** | **4.75** | **-2.45** | **2.30** | **100** | **1.00** | **GC WE; Pt CE; Ag/Ag^+^ RE; dried/N_2_-bubbled.** | ^[7]^ |
| **Imid.** | **[MMIM] bis(trifluoromethylsulfonyl)imide** | **4.61** | **-2.43** | **2.18** | **100** | **1.00** | **GC WE; Pt CE; Ag/Ag^+^ RE; dried/N_2_-bubbled.** | ^[7]^ |
| **Imid.** | **1-propyl-3-methylimidazolium bis(trifluoromethylsulfonyl)imide** | **4.56** | **-2.40** | **2.16** | **100** | **1.00** | **GC WE; Pt CE; Ag/Ag^+^ RE; dried/N_2_-bubbled.** | ^[7]^ |
| **Imid.** | **1-hydroxyethyl-2,3-dimethyl imidazolium tetrafluoroborate** | **5.95** | **-2.60** | **3.35** | **100** | **1.00** | **GC WE; Pt CE; Ag/Ag^+^ RE; dried/N_2_-bubbled.** | ^[7]^ |
| **Imid.** | **1-hydroxyethyl-2,3-dimethyl imidazolium perchlorate** | **5.95** | **-2.91** | **3.04** | **100** | **1.00** | **GC WE; Pt CE; Ag/Ag^+^ RE; dried/N_2_-bubbled.** | ^[7]^ |
| **Imid.** | **1-hydroxyethyl-2,3-dimethyl imidazolium nitrate** | **3.94** | **-2.34** | **1.60** | **100** | **1.00** | **GC WE; Pt CE; Ag/Ag^+^ RE; dried/N_2_-bubbled.** | ^[7]^ |
| **Imid.** | **1-hydroxyethyl-2,3-dimethyl imidazolium trifluoromethane sulfonate** | **5.18** | **-2.70** | **2.48** | **100** | **1.00** | **GC WE; Pt CE; Ag/Ag^+^ RE; dried/N_2_-bubbled.** | ^[7]^ |
| **Imid.** | **1-butyl-3-methylimidazolium tetrafluoroborate** | **5.18** | **-2.72** | **2.46** | **100** | **1.00** | **GC WE; Pt CE; Ag/Ag^+^ RE; dried/N_2_-bubbled.** | ^[7]^ |
| **Imid.** | **1-butyl-3-methylimidazolium trifluoromethane sulfonate** | **4.82** | **-2.71** | **2.11** | **100** | **1.00** | **GC WE; Pt CE; Ag/Ag^+^ RE; dried/N_2_-bubbled.** | ^[7]^ |
| **Imid.** | **1-butyl-3-methylimidazolium chloride** | **2.26** | **-2.72** | **-0.46** | **100** | **1.00** | **GC WE; Pt CE; Ag/Ag^+^ RE; dried/N_2_-bubbled.** | ^[7]^ |
| **Imid.** | **1-butyl-3-methylimidazolium hydrogen sulfate** | **4.25** | **-2.96** | **1.29** | **100** | **1.00** | **GC WE; Pt CE; Ag/Ag^+^ RE; dried/N_2_-bubbled.** | ^[7]^ |
| **Imid.** | **1-butyl-3-methylimidazolium iodide** | **2.02** | **-2.65** | **-0.63** | **100** | **1.00** | **GC WE; Pt CE; Ag/Ag^+^ RE; dried/N_2_-bubbled.** | ^[7]^ |
| **Imid.** | **1-butyl-3-methylimidazolium acetate** | **1.70** | **-2.58** | **-0.88** | **100** | **1.00** | **GC WE; Pt CE; Ag/Ag^+^ RE; dried/N_2_-bubbled.** | ^[7]^ |
| **Imid.** | **1-butyl-3-methylimidazolium bromide** | **2.20** | **-2.57** | **-0.47** | **100** | **1.00** | **GC WE; Pt CE; Ag/Ag^+^ RE; dried/N_2_-bubbled.** | ^[7]^ |
| **Imid.** | **1-butyl-3-methylimidazolium perchlorate** | **4.73** | **-2.57** | **2.16** | **100** | **1.00** | **GC WE; Pt CE; Ag/Ag^+^ RE; dried/N_2_-bubbled.** | ^[7]^ |
| **Imid.** | **1-butyl-3-methylimidazolium dicyanamide** | **1.68** | **-2.48** | **-0.80** | **100** | **1.00** | **GC WE; Pt CE; Ag/Ag^+^ RE; dried/N_2_-bubbled.** | ^[7]^ |
| **Imid.** | **1-butyl-3-methylimidazolium nitrate** | **1.81** | **-2.48** | **-0.67** | **100** | **1.00** | **GC WE; Pt CE; Ag/Ag^+^ RE; dried/N_2_-bubbled.** | ^[7]^ |
| **Imid.** | **1-butyl-3-methylimidazolium hexafluorophosphate** | **1.67** | **-2.51** | **-0.84** | **100** | **1.00** | **GC WE; Pt CE; Ag/Ag^+^ RE; dried/N_2_-bubbled.** | ^[7]^ |
| **Imid.** | **1-ethyl-3-methylimidazolium trifluoromethane sulfonate** | **4.37** | **-2.62** | **1.75** | **100** | **1.00** | **GC WE; Pt CE; Ag/Ag^+^ RE; dried/N_2_-bubbled.** | ^[7]^ |
| **Imid.** | **1-ethyl-3-methylimidazolium tetrafluoroborate** | **5.34** | **-3.15** | **2.19** | **100** | **1.00** | **GC WE; Pt CE; Ag/Ag^+^ RE; dried/N_2_-bubbled.** | ^[7]^ |
| **Imid.** | **1-hexyl-3-methylimidazolium tetrafluoroborate** | **5.32** | **-2.97** | **2.35** | **100** | **1.00** | **GC WE; Pt CE; Ag/Ag^+^ RE; dried/N_2_-bubbled.** | ^[7]^ |
| **Imid.** | **1-octyl-3-methylimidazolium tetrafluoroborate** | **5.56** | **-2.72** | **2.62** | **100** | **1.00** | **GC WE; Pt CE; Ag/Ag^+^ RE; dried/N_2_-bubbled.** | ^[7]^ |
| **Imid.** | **1-butyl-2,3-dimethyl imidazolium tetrafluoroborate** | **5.21** | **-2.99** | **2.22** | **100** | **1.00** | **GC WE; Pt CE; Ag/Ag^+^ RE; dried/N_2_-bubbled.** | ^[7]^ |
| **Imid.** | **1-ethyl-3-methylimidazolium ethyl sulfate** | **0.59** | **NR** | **NR** | **100** | **0.50** | **pNIPAAM ionogel; Au-IDEs; RT; stored 24 h before testing.** | ^[11]^ |
| **Imid.** | **pNIPAAM ionogel with 1-ethyl-3-methylimidazolium ethyl sulfate** | **1.47** | **NR** | **NR** | **100** | **0.50** | **pNIPAAM ionogel; Au-IDEs; RT; stored 24 h before testing.** | ^[11]^ |
| **Imid.** | **N-ethyl-N-methylimidazolium bis(fluorosulfonyl)imide** | **4.52** | **0.68** | **5.20** | **10** | **1.00** | **GC working electrode, Li/Li^+^ reference/counter electrode.** | ^[12]^ |
| **Morph.** | **4-(2-methoxyethyl)-4-methylmorpholinium tris(pentafluoroethyl)trifluorophosphate** | **3.98** | **-1.95** | **2.03** | **100** | **0.50** | **GC macro-electrode.** | ^[3]^ |
| **Morph.** | **N-methoxyethyl-N-methylmorpholinium bis(trifluoromethylsulfonyl)imide** | **4.68** | **-1.93** | **2.75** | **100** | **0.50** | **GC macro-electrode.** | ^[3]^ |
| **Morph.** | **N-methyl-N-(2-(2-methoxyethoxy)-ethyl)-morpholinium bis (trifluoromethylsulfonyl)imide** | **4.84** | **-2.59** | **2.25** | **2** | **NR** | **3 mm GC WE; Pt CE and RE; three-electrode cell.** | ^[13]^ |
| **Morph.** | **N-methyl-N-(2-(2-methoxyethoxy)-ethyl)-morpholinium bis-(trifluoromethylsulfonyl)imide (in acetonitrile)** | **5.48** | **-3.13** | **2.35** | **2** | **NR** | **0.906 M MeCN solution; 3 mm GC WE; Pt CE/RE.** | ^[13]^ |
| **Phos.** | **triethyl-n-pentylphosphonium bis(trifluoromethylsulfonyl)amide** | **≈5.4 to 5.5** | **-3.1 or -3.2** | **≈+2.3** | **10** | **NR** | **Glassy carbon (for limits) / Ni disk (for CV)** | ^[14]^ |
| **Phos.** | **triethyl-n-octylphosphonium bis(trifluoromethylsulfonyl)amide** | **≈5.4 to 5.5** | **-3.1 or -3.2** | **≈+2.3** | **10** | **NR** | **Glassy carbon (for limits) / Ni disk (for CV)** | ^[14]^ |
| **Phos.** | **triethyl(methoxymethyl)phosphonium bis(trifluoromethylsulfonyl)amide** | **≈5.3** | **-3.0** | **≈+2.3** | **10** | **NR** | **Glassy carbon (for limits) / Ni disk (for CV)** | ^[14]^ |
| **Phos.** | **triethyl(2-methoxyethyl)phosphonium bis(trifluoromethylsulfonyl)amide** | **≈5.4 to 5.5** | **-3.1 or -3.2** | **≈ +2.3** | **10** | **NR** | **Glassy carbon (for limits) / Ni disk (for CV)** | ^[14]^ |
| **Phos.** | **tris(n-hexyl)tetradecylphosphonium trifluorotris(pentafluoroethyl)phosphate** | **6.5 - 6.7** | **NR** | **NR** | **100** | **NR** | **0.1 M IL in Acetonitrile (Au/Pt microdisks).** | ^[2]^ |
| **Phos.** | **trihexyl(tetradecyl)phosphonium tris(pentafluoroethyl)trifluorophosphate** | **5.81** | **-3.64** | **2.17** | **100** | **0.50** | **GC macro-electrode.** | ^[3]^ |
| **Phos.** | **tris(P-hexyl)tetradecylphosphonium trifluorotris(pentafluoroethyl)phosphate** | **5.2** | **-2.7** | **2.5** | **100** | **1.00** | **Vacuum-dried, 1 mA cm^-2^ cutoff.** | ^[5]^ |
| **Phos.** | **trimethyl propyl phosphonium bis-fluorosulfonyl imide** | **5.05** | **-2.63** | **2.42** | **10** | **NR** | **Neat IL; 25 µm Pt ME; Ag-wire CE/QRE; vs Ag/Ag^+^; RT.** | ^[15]^ |
| **Phos.** | **trimethyl isobutyl phosphonium bis-fluorosulfonyl imide** | **5.43** | **-2.90** | **2.53** | **10** | **NR** | **Neat IL; 25 µm Pt ME; Ag-wire CE/QRE; vs Ag/Ag^+^; RT.** | ^[15]^ |
| **Phos.** | **trimethyl propyl phosphonium bis-fluorosulfonyl imide with LiPF6** | **6.25** | **-3.83** | **2.42** | **10** | **NR** | **0.5 M LiPF_6_ in IL; 25 µm Pt ME; Ag CE/QRE; RT; neat IL solid at RT.** | ^[15]^ |
| **Phos.** | **trimethyl isobutyl phosphonium bis-fluorosulfonyl imide with LiPF6** | **6.33** | **-3.80** | **2.53** | **10** | **NR** | **0.5 M LiPF_6_ in IL; 25 µm Pt ME; Ag CE/QRE; RT; neat IL solid at RT.** | ^[15]^ |
| **Phos.** | **trimethyl isobutyl phosphonium bis-trifluoromethylsulfonyl imide with LiPF6** | **6.30** | **-3.80** | **2.50** | **10** | **NR** | **0.5 M LiPF_6_ in IL; 25 µm Pt ME; Ag CE/QRE; RT; neat IL solid at RT.** | ^[15]^ |
| **Phos.** | **trimethyl(isobutyl)phosphonium bis(fluorosulfonyl)imide** | **6.0** | **-3.5** | **2.5** | **20** | **NR** | **Neat IL; 1 mm GC WE; Pt CE; Ag/AgOTf RE; 25 °C.** | ^[16]^ |
| **Phos.** | **trihexyl(tetradecyl)phosphonium dicyanamide** | **5.12** | **NR** | **NR** | **100** | **0.50** | **pNIPAAM ionogel; Au-IDEs; RT; stored 24 h before testing.** | ^[11]^ |
| **Phos.** | **pNIPAAM ionogel with trihexyl(tetradecyl)phosphonium dicyanamide** | **3.88** | **NR** | **NR** | **100** | **0.50** | **pNIPAAM ionogel; Au-IDEs; RT; stored 24 h before testing.** | ^[11]^ |
| **Phos.** | **Symmetrically substituted cyclic phosphonium (R/R' = methyl)** | **6.73** | **0.56** | **7.29** | **NR** | **NR** | **DFT-derived; M06-L/SMD; vs Li^+^/Li.** | ^[17]^ |
| **Phos.** | **Symmetrically substituted cyclic phosphonium (R/R' = ethyl)** | **6.22** | **0.82** | **7.05** | **NR** | **NR** | **DFT-derived; M06-L/SMD; vs Li^+^/Li.** | ^[17]^ |
| **Phos.** | **Symmetrically substituted cyclic phosphonium (R/R' = propyl)** | **6.05** | **0.77** | **6.82** | **NR** | **NR** | **DFT-derived; M06-L/SMD; vs Li^+^/Li.** | ^[17]^ |
| **Phos.** | **Symmetrically substituted cyclic phosphonium (R/R' = butyl)** | **5.83** | **0.71** | **6.55** | **NR** | **NR** | **DFT-derived; M06-L/SMD; vs Li^+^/Li.** | ^[17]^ |
| **Phos.** | **Symmetrically substituted cyclic phosphonium (R/R' = pentyl)** | **5.58** | **0.76** | **6.34** | **NR** | **NR** | **DFT-derived; M06-L/SMD; vs Li^+^/Li.** | ^[17]^ |
| **Phos.** | **Symmetrically substituted cyclic phosphonium (R/R' = hexyl)** | **5.42** | **0.75** | **6.17** | **NR** | **NR** | **DFT-derived; M06-L/SMD; vs Li^+^/Li.** | ^[17]^ |
| **Phos.** | **Symmetrically substituted cyclic phosphonium (R/R' = phenyl)** | **5.10** | **0.71** | **5.81** | **NR** | **NR** | **DFT-derived; M06-L/SMD; vs Li^+^/Li.** | ^[17]^ |
| **Phos.** | **Symmetrically substituted cyclic phosphonium (R/R' = napthyl)** | **4.09** | **0.75** | **4.84** | **NR** | **NR** | **DFT-derived; M06-L/SMD; vs Li^+^/Li.** | ^[17]^ |
| **Phos.** | **Symmetrically substituted cyclic phosphonium (R/R' = diphenyl ether)** | **4.30** | **0.62** | **4.92** | **NR** | **NR** | **DFT-derived; M06-L/SMD; vs Li^+^/Li.** | ^[17]^ |
| **Phos.** | **Symmetrically substituted cyclic phosphonium (R/R' = dimethyl ether)** | **4.57** | **1.36** | **5.93** | **NR** | **NR** | **DFT-derived; M06-L/SMD; vs Li^+^/Li.** | ^[17]^ |
| **Phos.** | **Symmetrically substituted cyclic phosphonium (R/R' = methoxy 1,4-dioxane)** | **4.23** | **1.37** | **5.60** | **NR** | **NR** | **DFT-derived; M06-L/SMD; vs Li^+^/Li.** | ^[17]^ |
| **Phos.** | **Symmetrically substituted cyclic phosphonium (R/R' = methoxy benzene)** | **3.48** | **1.43** | **4.90** | **NR** | **NR** | **DFT-derived; M06-L/SMD; vs Li^+^/Li.** | ^[17]^ |
| **Phos.** | **Symmetrically substituted cyclic phosphonium (R/R' = methoxy ethene)** | **3.84** | **1.39** | **5.23** | **NR** | **NR** | **DFT-derived; M06-L/SMD; vs Li^+^/Li.** | ^[17]^ |
| **Phos.** | **Symmetrically substituted cyclic phosphonium (R/R' = methoxy terbutyl)** | **4.44** | **1.26** | **5.70** | **NR** | **NR** | **DFT-derived; M06-L/SMD; vs Li^+^/Li.** | ^[17]^ |
| **Phos.** | **Symmetrically substituted cyclic phosphonium (R/R' = NH2)** | **5.90** | **0.36** | **6.27** | **NR** | **NR** | **DFT-derived; M06-L/SMD; vs Li^+^/Li.** | ^[17]^ |
| **Phos.** | **Symmetrically substituted cyclic phosphonium (R/R' = dimethylamine)** | **4.08** | **1.12** | **5.20** | **NR** | **NR** | **DFT-derived; M06-L/SMD; vs Li^+^/Li.** | ^[17]^ |
| **Phos.** | **Symmetrically substituted cyclic phosphonium (R/R' = N-methyl-phenylamine)** | **2.90** | **1.81** | **4.70** | **NR** | **NR** | **DFT-derived; M06-L/SMD; vs Li^+^/Li.** | ^[17]^ |
| **Phos.** | **Symmetrically substituted cyclic phosphonium (R/R' = N-methyl-paraamino-phenylamine)** | **1.78** | **-** | **-** | **NR** | **NR** | **DFT-derived; M06-L/SMD; vs Li^+^/Li.** | ^[17]^ |
| **Phos.** | **Symmetrically substituted cyclic phosphonium (R/R' = para amino-phenyl)** | **3.85** | **0.39** | **4.24** | **NR** | **NR** | **DFT-derived; M06-L/SMD; vs Li^+^/Li.** | ^[17]^ |
| **Phos.** | **Symmetrically substituted cyclic phosphonium (R/R' = ortho diamino-phenyl)** | **3.37** | **0.71** | **4.08** | **NR** | **NR** | **DFT-derived; M06-L/SMD; vs Li^+^/Li.** | ^[17]^ |
| **Phos.** | **Symmetrically substituted cyclic phosphonium (R/R' = meta diamino-phenyl)** | **3.40** | **0.53** | **3.93** | **NR** | **NR** | **DFT-derived; M06-L/SMD; vs Li^+^/Li.** | ^[17]^ |
| **Phos.** | **Symmetrically substituted cyclic phosphonium (R/R' = ortho,para triamino-phenyl)** | **3.64** | **0.32** | **3.96** | **NR** | **NR** | **DFT-derived; M06-L/SMD; vs Li^+^/Li.** | ^[17]^ |
| **Pip.** | **N-methyl-N'-propylpiperidinium bis(trifluoromethylsulfonyl)imide** | **5.8** | **-3.2** | **2.7** | **50** | **0.50** | **GC electrode.** | ^[1]^ |
| **Pip.** | **1-(3-methoxypropyl)-1-methylpiperidinium bis(trifluoromethylsulfonyl)imide** | **4.31** | **-1.94** | **2.37** | **100** | **0.50** | **GC macro-electrode.** | ^[3]^ |
| **Pip.** | **1-(2-methoxyethyl)-1-methylpiperidinium tris(pentafluoroethyl)trifluorophosphate** | **3.87** | **-1.75** | **2.12** | **100** | **0.50** | **GC macro-electrode.** | ^[3]^ |
| **Pip.** | **1-methyl-1-propylpiperidinium bis(trifluoromethylsulfonyl)imide** | **7.06** | **-3.06** | **4.00** | **NR** | **1.00** | **Tested at 1.0 mA cm^-2^ cutoff.** | ^[9]^ |
| **Pip.** | **1-butyl-1-methylpiperidinium bis(trifluoromethylsulfonyl)imide** | **6.86** | **-3.10** | **3.76** | **NR** | **1.00** | **Tested at 1.0 mA cm^-2^ cutoff.** | ^[9]^ |
| **Pip.** | **1-butyl-1-methylpiperidinium bis(trifluoromethylsulfonyl)imide** | **5.57** | **-3.55** | **3.02** | **100** | **1.00** | **GC WE; Pt CE; Ag/Ag^+^ RE; dried/N_2_-bubbled.** | ^[7]^ |
| **Pip.** | **N-methylpiperidinium-N-acetate bis(trifluoromethylsulfonyl)imide** | **≈5.0** | **-2.45** | **2.55** | **5** | **NR** | **GC WE; Pt CE; Ag RE; Ar; vs Fc/Fc^+^.** | ^[18]^ |
| **Pyraz.** | **1,2-dimethyl-4-fluoropyrazolium tetrafluoroborate** | **4.0** | **-1.4** | **2.6** | **50** | **0.50** | **GC electrode with Ag QRE.** | ^[1]^ |
| **Pyraz.** | **1-ethyl-2-methylpyrazolium tetrafluoroborate** | **4.4** | **1.2** | **5.5** | **50** | **0.50** | **Pt electrode with Li QRE.** | ^[1]^ |
| **Pyrid.** | **p-dimethylamino-N-butylPyridinium** | **2.1** | **-0.5** | **1.7** | **200** | **NR** | **GC working electrode.** | ^[1]^ |
| **Pyrid.** | **p-dimethylamino-N-butylPyridinium** | **2.6** | **-1.7** | **0.9** | **200** | **NR** | **GC working electrode.** | ^[1]^ |
| **Pyrid.** | **N-butylPyridinium** | **2.0** | **-0.1** | **1.9** | **100** | **NR** | **GC working electrode.** | ^[1]^ |
| **Pyrid.** | **N-butylPyridinium bis(trifluoromethylsulfonyl)imide** | **3.9** | **-1.3** | **2.5** | **50** | **0.50** | **GC electrode.** | ^[1]^ |
| **Pyrid.** | **p-dimethylamino-N-butylPyridinium bis(trifluoromethylsulfonyl)imide** | **3.5** | **-2.0** | **1.5** | **50** | **0.50** | **GC electrode.** | ^[1]^ |
| **Pyrid.** | **N-butyl-3-methyl-Pyridinium dicyanamide** | **2.96** | **-1.25** | **1.71** | **100** | **0.50** | **GC macro-electrode.** | ^[3]^ |
| **Pyrid.** | **N-hexylPyridinium bis(trifluoromethylsulfonyl)imide** | **3.96** | **-1.22** | **2.74** | **100** | **0.50** | **GC macro-electrode.** | ^[3]^ |
| **Pyrid.** | **N-(3-hydroxypropyl)Pyridinium bis(trifluoromethylsulfonyl)imide** | **3.81** | **-1.12** | **2.69** | **100** | **0.50** | **GC macro-electrode.** | ^[3]^ |
| **Pyrid.** | **N-butylPyridinium bis(trifluoromethylsulfonyl)imide** | **4.37** | **-1.54** | **2.83** | **100** | **1.00** | **GC WE; Pt CE; Ag/Ag^+^ RE; dried/N_2_-bubbled.** | ^[7]^ |
| **Pyrid.** | **N-butylPyridinium tetrafluoroborate** | **6.13** | **-2.48** | **3.65** | **100** | **1.00** | **GC WE; Pt CE; Ag/Ag^+^ RE; dried/N_2_-bubbled.** | ^[7]^ |
| **Pyrid.** | **N-butylPyridinium hydrogen sulfate** | **5.44** | **-2.20** | **3.24** | **100** | **1.00** | **GC WE; Pt CE; Ag/Ag^+^ RE; dried/N_2_-bubbled.** | ^[7]^ |
| **Pyrid.** | **N-butylPyridinium nitrate** | **3.55** | **-1.57** | **1.78** | **100** | **1.00** | **GC WE; Pt CE; Ag/Ag^+^ RE; dried/N_2_-bubbled.** | ^[7]^ |
| **Pyrid.** | **Pyridinium bisulphate** | **2.1** | **-0.1** | **2.0** | **50** | **NR** | **Pt WE; Ag-wire QRE; tested at 303 K.** | ^[19]^ |
| **Pyrid.** | **Pyridinium bisulphate** | **2.3** | **-0.5** | **1.8** | **50** | **NR** | **Au WE; Ag-wire QRE; tested at 303 K.** | ^[19]^ |
| **Pyrid.** | **Pyridinium sulphate** | **2.3** | **-0.1** | **2.2** | **50** | **NR** | **Pt WE; Ag-wire QRE; tested at 303 K.** | ^[19]^ |
| **Pyrid.** | **Pyridinium sulphate** | **2.4** | **-0.5** | **1.9** | **50** | **-** | **Au WE; Ag-wire QRE; tested at 303 K.** | ^[19]^ |
| **Pyrrol.** | **N-methyl-N'-propylpyrrolidinium bis(trifluoromethylsulfonyl)amide** | **4.7** | **-1.9** | **2.8** | **100** | **0.50** | **Pt electrode.** | ^[1]^ |
| **Pyrrol.** | **N-methyl-N'-propylpyrrolidinium p-toluenesulfonate** | **2.9** | **-1.3** | **1.6** | **100** | **0.10** | **Pt electrode.** | ^[1]^ |
| **Pyrrol.** | **1-hexyl-1-methyl-pyrrolidinium bis(trifluoromethylsulfonyl)imide** | **5.67** | **-3.06** | **2.61** | **100** | **0.50** | **GC macro-electrode.** | ^[3]^ |
| **Pyrrol.** | **1-butyl-1-methylpyrrolidinium trifluoromethanesulfonate** | **4.83** | **-2.95** | **1.88** | **100** | **0.50** | **GC macro-electrode.** | ^[3]^ |
| **Pyrrol.** | **1-butyl-1-methylpyrrolidinium dicyanamide** | **4.62** | **-2.95** | **1.67** | **100** | **0.50** | **GC macro-electrode.** | ^[3]^ |
| **Pyrrol.** | **1-butyl-1-methylpyrrolidinium bis(trifluoromethylsulfonyl)imide** | **5.20** | **-2.92** | **2.28** | **100** | **0.50** | **GC macro-electrode.** | ^[3]^ |
| **Pyrrol.** | **1-butyl-1-methylpyrrolidinium trifluoroacetate** | **4.20** | **-2.85** | **1.35** | **100** | **0.50** | **GC macro-electrode (measured at 35 °C).** | ^[3]^ |
| **Pyrrol.** | **butylmethylpyrrolidinium bis(trifluoromethylsulfonium)imide / N-butyl-N-methyl-pyrrolidinium bis(trifluoromethylsulfonyl)imide** | **5.9** | **-** | **-** | **100** | **1.91** | **Pt thin-film, <1 RH%, 15 µA cutoff.** | ^[4]^ |
| **Pyrrol.** | **butylmethylpyrrolidinium tris(pentafluoroethyl)trifluorophosphate** | **6.5** | **-** | **-** | **100** | **1.91** | **Pt thin-film, <1 RH%, 15 µA cutoff.** | ^[4]^ |
| **Pyrrol.** | **N-butyl-N-methyl-pyrrolidinium bis(trifluoromethylsulfonyl)imide** | **4.2** | **-2.8** | **1.4** | **100** | **1.00** | **Vacuum-dried, 1 mA cm^-2^ cutoff.** | ^[5]^ |
| **Pyrrol.** | **N-butyl-N-methyl pyrrolidinium bis(trifluoromethylsulfonyl)imide** | **6.55** | **-3.50** | **3.05** | **100** | **1.00** | **GC WE; Pt CE; Ag/Ag^+^ RE; dried/N₂-bubbled.** | ^[7]^ |
| **Pyrrol.** | **N-methyl-N-propylpyrrolidinium bis(fluorosulfonyl)imide** | **>4.85** | **<0.40** | **>5.25** | **10** | **1.00** | **GC WE; Li/Li^+^ RE/CE; 0.4–5.25 V stable.** | ^[12]^ |
| **Pyrrol.** | **N,N-dimethylpyrrolidinium tetrafluoroborate** | **6.02** | **-1.23** | **-7.253** | **-** | **-** | **DFT; M06L/6-311++G(d,p).** | ^[20]^ |
| **Pyrrol.** | **N-hexyl-N-methylpyrrolidinium tetrafluoroborate** | **6.16** | **-1.10** | **-7.267** | **-** | **-** | **DFT; M06L/6-311++G(d,p).** | ^[20]^ |
| **Pyrrol.** | **N,N-dimethylpyrrolidinium hexafluorophosphate** | **6.43** | **-1.36** | **-7.793** | **-** | **-** | **DFT; M06L/6-311++G(d,p).** | ^[20]^ |
| **Pyrrol.** | **N,N-dimethylpyrrolidinium bis(trifluoromethylsulfonyl)imide** | **5.26** | **-1.27** | **-6.535** | **-** | **-** | **DFT; M06L/6-311++G(d,p).** | ^[20]^ |
| **Pyrrol.** | **N,N-dimethylpyrrolidinium trifluoromethanesulfonate** | **4.51** | **-1.15** | **-5.662** | **-** | **-** | **DFT; M06L/6-311++G(d,p).** | ^[20]^ |
| **Sulf.** | **triethylsulfonium bis(trifluoromethylsulfonyl)imide** | **4.8** | **-2.1** | **2.7** | **50** | **0.50** | **GC electrode.** | ^[1]^ |
| **Sulf.** | **tributylsulfonium bis(trifluoromethylsulfonyl)imide** | **4.9** | **-2.3** | **2.6** | **50** | **0.50** | **GC electrode.** | ^[1]^ |
| **Sulf.** | **triethylsulfonium bis(trifluoromethylsulfonyl)imide** | **3.51** | **-1.30** | **2.21** | **100** | **0.50** | **GC macro-electrode.** | ^[3]^ |
| **Sulf.** | **diethylmethylsulfonium bis(trifluoromethylsulfonyl)imide** | **5.0** | **-** | **-** | **100** | **1.91** | **Pt thin-film, <1 RH%,** | ^[4]^ |
| **Sulf.** | **triethylsulfonium bis(trifluoromethylsulfonyl)imide** | **5.02** | **-2.24** | **2.78** | **-** | **1.00** | **Tested at 1.0 mA cm^-2^cutoff.** | ^[9]^ |
| **Sulf.** | **S-butyltetrahydrothiophenium bis((trifluoromethyl)sulfonyl)imide** | **4.41** | **-2.09** | **2.32** | **-** | **-** | **GC WE; Ag[OTf]/Ag RE; vs Fc⁺/Fc; RT** | ^[21]^ |
| **Sulf.** | **S-(2-methoxyethyl)tetrahydrothiophenium bis((trifluoromethyl)sulfonyl)imide** | **4.42** | **-2.15** | **2.27** | **-** | **-** | **GC WE; Ag[OTf]/Ag RE; vs Fc⁺/Fc; RT** | ^[21]^ |
| **Sulf.** | **dimethyl(2-methoxyethyl)sulfonium bis(trifluoromethylsulfonyl)imide** | **3.75** | **1.34** | **5.09** | **-** | **-** | **GC WE; Li RE; 20 mg mL^-1^ Li[NTf_2_].** | ^[22]^ |
| **Sulf.** | **dimethyl(2-(2-methoxyethoxy)ethyl)sulfonium bis(trifluoromethylsulfonyl)imide** | **3.57** | **1.37** | **4.94** | **-** | **-** | **GC WE; Li RE; 20 mg mL^-1^ Li[NTf_2_].** | ^[22]^ |
| **Sulf.** | **ethylmethyl(2-methoxyethyl)sulfonium bis(trifluoromethylsulfonyl)imide** | **3.87** | **1.24** | **5.11** | **-** | **-** | **GC WE; Li RE; 20 mg mL^-1^ Li[NTf_2_].** | ^[22]^ |
| **Sulf.** | **di(2-methoxyethyl)methylsulfonium bis(trifluoromethylsulfonyl)imide** | **3.39** | **1.36** | **4.75** | **-** | **-** | **GC WE; Li RE; 20 mg mL^-1^ Li[NTf_2_].** | ^[22]^ |
| **Sulf.** | **di[2-(2-methoxyethoxy)ethyl]methylsulfonium bis(trifluoromethylsulfonyl)imide** | **3.33** | **1.36** | **4.69** | **-** | **-** | **GC WE; Li RE; 20 mg mL^-1^ Li[NTf_2_].** | ^[22]^ |

***Note****: EW = electrochemical window; App. EW = apparent electrochemical window; E_cat_ = cathodic stability limit; E_an_ = anodic stability limit; ν = scan rate; j_cut_ = cutoff current density. Cation-family abbreviations: Amm. = ammonium; Imid. = imidazolium; Morph. = morpholinium; Phos. = phosphonium; Pip. = piperidinium; Pyraz. = pyrazolium; Pyrid. = pyridinium; Pyrrol. = pyrrolidinium; Sulf. = sulfonium. WE = working electrode; CE = counter electrode; RE = reference electrode; QRE = quasi-reference electrode; ME = microelectrode; GC = glassy carbon; Au-IDEs = gold interdigitated electrodes; GF = glass fibre; RT = room temperature; NR = not reported; MeCN = acetonitrile. Reported EW values are protocol-dependent and should be compared only as literature-derived apparent stability windows.*

**Table S2**: Comparative electrochemical and transport performance of representative phosphonium IL electrolytes in Li and post-Li battery systems. Representative ionic conductivities (σ), viscosities (η), and apparent ESW are summarized from literature reports. Because electrolyte composition, salt concentration, reference electrode, cutoff current, temperature, and cell configuration differ among reports, the values should be interpreted as representative ranges rather than directly comparable intrinsic properties.

| System | Representative phosphonium IL electrolyte | σ  (mS cm⁻¹) | η  (mPa s) | App. ESW (V) | Ref |
| --- | --- | --- | --- | --- | --- |
| Li | **[P_1,1,1_i_4_][FSI] + NaFSI** | **0.8 – 4.4** | **60 – 320** | **≈6.0** | ^[16]^ |
| Na | **[P_2,2,2_,(1O1)][FSI]** | **3.7 – 8.5** | **66 – 78** | **4.8–5.0** | ^[23]^ |
| Zn | **Functionalized phosphonium-cation aqueous Zn electrolyte** | **NR** | **NR** | **4.5–5.0** | ^[24]^ |
| Mg | **[P_6,6,6,14_]-based IL/DES electrolyte** | **0.5 – 1** | **150 – 250** | **4.5–4.8** | ^[25]^ |
| Al | **Phosphonium-based IL/DES electrolyte** | **0.2 – 0.5** | **250 – 400** | **4.7–5.2** | ^[26, 27]^ |
| Supercapacitor | **[P_6,6,6,14_][TFSI]/MeCN; PSNBF_4_/AN or PN** | **27.5 – 49.8** | **low–moderate** | **>4.7 (electrolyte); device 3.1–3.4** | ^[28, 29]^ |
| Hybrid gel/DES | **[P_1,1,1_i_4_][FSI]/LiFSI/PEGDA or phosphonium polymer electrolyte** | **0.2 – 3.0** | **50 – 120** | **3.2 – 4.5** | ^[30, 31]^ |

**Representative values are extracted from the original reports measured under different electrolyte compositions, salt concentrations, temperatures, electrode configurations, and testing protocols. Therefore, the values should be interpreted as representative ranges in the literature rather than as directly comparable intrinsic properties. NR = not reported.*

**References**

1.	Matsumoto, H., *Electrochemical Windows of Room-Temperature Ionic Liquids (RTILs).* Electrochemical Aspects of Ionic Liquids, 2011: p. 43–63.10.1002/9781118003350.ch4

2.	Buzzeo, M.C., C. Hardacre, and R.G. Compton, *Extended electrochemical windows made accessible by room temperature ionic liquid/organic solvent electrolyte systems.* Chemphyschem, 2006. **7**(1): p. 176–180.10.1002/cphc.200500361

3.	Hayyan, M., et al., *Investigating the electrochemical windows of ionic liquids.* Journal of Industrial and Engineering Chemistry, 2013. **19**(1): p. 106–112.<https://doi.org/10.1016/j.jiec.2012.07.011>

4.	Doblinger, S., T.J. Donati, and D.S. Silvester, *Effect of Humidity and Impurities on the Electrochemical Window of Ionic Liquids and Its Implications for Electroanalysis.* The Journal of Physical Chemistry C, 2020. **124**(37): p. 20309–20319.10.1021/acs.jpcc.0c07012

5.	O’Mahony, A.M., et al., *Effect of Water on the Electrochemical Window and Potential Limits of Room-Temperature Ionic Liquids.* Journal of Chemical & Engineering Data, 2008. **53**(12): p. 2884–2891.10.1021/je800678e

6.	Zhao, C., et al., *Electrochemistry of Room Temperature Protic Ionic Liquids.* The Journal of Physical Chemistry B, 2008. **112**(23): p. 6923–6936.10.1021/jp711804j

7.	Li, Q., et al., *The electrochemical stability of ionic liquids and deep eutectic solvents.* Science China Chemistry, 2016. **59**(5): p. 571–577.10.1007/s11426-016-5566-3

8.	Jeong, S., et al., *Asymmetric ammonium-based ionic liquids as electrolyte components for safer, high-energy, electrochemical storage devices.* Energy Storage Materials, 2019. **18**: p. 1–9.<https://doi.org/10.1016/j.ensm.2019.01.015>

9.	Gancarz, P., E. Zorębski, and M. Dzida, *Influence of experimental conditions on the electrochemical window. Case study on bis(trifluoromethylsulfonyl)imide-based ionic liquids.* Electrochemistry Communications, 2021. **130**: p. 107107.<https://doi.org/10.1016/j.elecom.2021.107107>

10.	Lerch, S. and T. Strassner, *Expanding the Electrochemical Window: New Tunable Aryl Alkyl Ionic Liquids (TAAILs) with Dicyanamide Anions.* Chemistry – A European Journal, 2019. **25**(71): p. 16251–16256.<https://doi.org/10.1002/chem.201902797>

11.	Gil-González, N., et al., *Electrical and electrochemical properties of imidazolium and phosphonium-based pNIPAAM ionogels.* Electrochimica Acta, 2020. **345**: p. 136167.<https://doi.org/10.1016/j.electacta.2020.136167>

12.	Kim, H.-T., et al., *Novel Pyrrolinium-based Ionic Liquids for Lithium Ion Batteries: Effect of the Cation on Physicochemical and Electrochemical Properties.* Electrochimica Acta, 2017. **240**: p. 267–276.<https://doi.org/10.1016/j.electacta.2017.04.078>

13.	Lunin, V.V., et al., *Structural and thermoanalytical features of novel morpholinium-based high-voltage ionic liquid.* Structural Chemistry, 2017. **28**(2): p. 279–287.10.1007/s11224-016-0768-z

14.	Lucht, B.L., et al., *Electrochemical Interfaces in Electrochemical Energy Storage Systems.* Journal of The Electrochemical Society, 2015. **162**(13): p. Y13.10.1149/2.0171513jes

15.	Salem, N., et al., *Physical and Electrochemical Properties of Some Phosphonium-Based Ionic Liquids and the Performance of Their Electrolytes in Lithium-Ion Batteries.* Journal of the Electrochemical Society, 2017. **164**(8): p. H5202–H5209.10.1149/2.0061708jes

16.	Girard, G.M.A., et al., *Electrochemical and physicochemical properties of small phosphonium cation ionic liquid electrolytes with high lithium salt content.* Physical Chemistry Chemical Physics, 2015. **17**(14): p. 8706–8713.10.1039/C5CP00205B

17.	Dathar, G.K.P., et al., *Electrochemical Stability of Functionalized Cyclic Phosphonium (CylP+nA−) Ionic Liquid Based Battery Electrolytes.* Journal of the Electrochemical Society, 2016. **163**(6): p. A1057–A1063.10.1149/2.1131606jes

18.	Dong, T., et al., *A piperidinium-based ionic liquid electrolyte to enhance the electrochemical properties of LiFePO4 battery.* Ionics, 2015. **21**(8): p. 2109–2117.10.1007/s11581-015-1388-0

19.	Muhammad, S., et al., *Moisture Sorption by Low-Cost Pyridinium-Based Protic Ionic Liquids: Kinetics and Physico-Electrochemical Properties.* ChemistryOpen, 2025. **14**(1): p. e202400165.<https://doi.org/10.1002/open.202400165>

20.	Asha, S., K.P. Vijayalakshmi, and B.K. George, *Electronic structural studies of pyrrolidinium-based ionic liquids for electrochemical application.* International Journal of Quantum Chemistry, 2019. **119**(17): p. e25972.<https://doi.org/10.1002/qua.25972>

21.	Neale, A.R., et al., *An ether-functionalised cyclic sulfonium based ionic liquid as an electrolyte for electrochemical double layer capacitors.* Journal of Power Sources, 2016. **326**: p. 549–559.<https://doi.org/10.1016/j.jpowsour.2016.06.085>

22.	Coadou, E., et al., *Synthesis and Thermophysical Properties of Ether-Functionalized Sulfonium Ionic Liquids as Potential Electrolytes for Electrochemical Applications.* ChemPhysChem, 2016. **17**(23): p. 3992–4002.<https://doi.org/10.1002/cphc.201600882>

23.	Ferdousi, S.A., et al., *High-Performance Cycling of Na Metal Anodes in Phosphonium and Pyrrolidinium Fluoro(sulfonyl)imide Based Ionic Liquid Electrolytes.* ACS Applied Materials & Interfaces, 2022. **14**(13): p. 15784–15798.10.1021/acsami.1c24812

24.	Ma, L., et al., *Functionalized Phosphonium Cations Enable Zinc Metal Reversibility in Aqueous Electrolytes.* Angewandte Chemie International Edition, 2021. **60**(22): p. 12438–12445.<https://doi.org/10.1002/anie.202017020>

25.	Tomich, A.W., et al., *Electrolyte Engineering with Carboranes for Next-Generation Mg Batteries.* ACS Central Science, 2024. **10**(2): p. 264–271.10.1021/acscentsci.3c01176

26.	Matuszek, K., et al., *Unexpected Energy Applications of Ionic Liquids.* Advanced Materials, 2024. **36**(23): p. 2313023.<https://doi.org/10.1002/adma.202313023>

27.	Liu, T., et al., *The effect of Fe-anion interactions on corrosion of ferrous surfaces by phosphonium ionic liquids.* Corrosion Science, 2024. **227**: p. 111734.<https://doi.org/10.1016/j.corsci.2023.111734>

28.	Nguyen, H.V.T., et al., *Implementation of phosphonium salt for high-performance supercapacitors from room to ultra-low temperature conditions.* Journal of Energy Storage, 2025. **106**: p. 114750.<https://doi.org/10.1016/j.est.2024.114750>

29.	Frackowiak, E., G. Lota, and J. Pernak, *Room-temperature phosphonium ionic liquids for supercapacitor application.* Applied Physics Letters, 2005. **86**(16).10.1063/1.1906320

30.	Rawat, S., et al., *Polyethylene Oxide Incorporated Ammonium Iodide Doped With Ionic Liquid Trihexyl (Tetradecy) Phosphonium Dicyanamide-Based Polymer Electrolyte for Dual Energy Storage Devices.* Energy Storage, 2025. **7**(1).<https://doi.org/10.1002/est2.70107>

31.	Ghorpade, P.R., et al., *Optimizing Phosphonium-Based Ionic Liquid Electrolytes for Enhanced Supercapacitor Performance.* Journal of Chemical & Engineering Data, 2025. **70**(7): p. 2741–2756.10.1021/acs.jced.4c00751
